# Supplementary material for: Mobilome of the Rhus Gall Aphid Schlechtendalia chinensis Provides Insight into TE Insertion-Related Inactivation of Functional Genes
Source: Int J Mol Sci. 2022 Dec 15;23(24):15967. doi: 10.3390/ijms232415967 (PMC9783078; doi:10.3390/ijms232415967)
Supplement: Supplementary file 1 [file ijms-23-15967-s001.zip › Supplementary Data S6.pdf]

Mariner transcripts

>F01_transcript_762

AUGGCCGGCAGCACCACCAGCCGCGAGAGCACCGCCGCGGACGAAGAACGACCGUUGCCG
GUGUCGGUGAACAAGUACAAAAAAAAGAAACCUUUGCCCGCGACCAGGCCCAAAGAACCG
CAACCGUCGGCCGAAGUACAAUUGGACGUGACGCCGAGCCCGUCAACGGUUACCACGACC
GCGCCACCGCACCCACUGUCGCAACCGAGUCCCGAUACGACGGCGGCGACGGACACACGG
GCAAAGACCAAAGCCAAGUAUGGGAACGGUACGCGGCCGCGGUUCAGCAUCAAAGACUAC
AAAACCAGUACCACGUCGACGACCACCAGCACGGAGAAAGCGCCGUCUGGUCGACGGACG
ACGACGACGACGUCGACCGCGGUGCCGGAAACGGUUGUAGAACAGCAAACCGCGAGGAAA
CGGUACAGACCGCGGCCGAACCGGUACAAGACCACCACUUCCACCACCGCCACCGCCACCG
CCACCGCCGCCACCGCCGCCACCGCCGCCACAGAGGAACAGCAACGGCAGGAAACCGCGAC
CGCGGCGGCGUACCGUAGCAAGUACAAGCCCGGCGGCAAGUAUCACAACAGGUUGAGGAC
GAGCACCGAGUCGGCGGACGAGGGCGUCGAAUCGGCCACGGAGCCGACCAGACCGAUGGC
CGUGUACUCGGCCAAACGGCGGACGGUGCCGACCGCGAGUACCGCCGACGAGCACAACGC
GGUCAGGAGGAGCACGUCGACCACGUCGCCGCUCGACGACGACGUCACCCCGCCCGAGCC
GUUGGACGCCGCGUCAUCCGAGGACGUCGACUAUGUCGCUACCACGGUCGCGGUGACCAU
GGCGGUCGCUCCGGCCAGACACAAGUACCAUACUUCGUACGCGGCCGAACGACCGCUGUU
GCCCAUCGAAUCGUUCUUCCAGUCGUCGAUGGCCGCCUCUAAACGGUAUCACAAUCACAA
CAAACGGCGAUGA

>F01_transcript_1320

AUGAACAACAUAGAUGAUAAAAUAGGUAAUGGCAAUAAUAAUCAACAACCACAGGCACC
UACAAAUAAUGAUAAAAUAAUAGCUGUUAAAUCAGCAGAAGAUUCACAGGUACAUCAUA
AAAACAAUAAUAAUGAAAAAAAAAAAGGAAACGCCUUAUUAACAGAUGAAGAAUCAAUA
AAUGUGCGUCUACUGGGUAAACCAUUAGUUUCACGUGGCCAUUAUACAUUUUAUAGUGC
UUUUGCAUACCGAAAACAUGGUGACUUAAUGAAACAGAAAAAUGGAAGACUGUGGGAAG
GUGAUAAAAAUGUUACAGAUACCAAAUCAUGUAAUCUAUUGUCUUCUUGUUGCUCUAAU
GGUAACAAUGGUUUGUGUUCCUGUGAUUUCGGCUCCUCCUCGUCAUCAUCGUUAACCUGU
UCUGAAUGGUCUGUUGUGCACAUGAAUCAUUUUUAUGCAGUUCGCCCUUGGUAUAGUAA
ACAAAACAGUAGUGAUCAACGGAAAUCAUGGCAACCUCAUCAAAAACCUUCACGAAAAU
UGACAUCAACAGCUGUGGAACAGCAAUCAGUAUGUAUAGCAGAAUUGGAGUUACUGUGG
CAUGAUGAUCAAAUAUCAAUGUCAAACAUAAUACCAUUGUCGACAUCAUCUUGUAAGAA
ACGUGGUAGAAAUAAAGCUGUGCCAUCAUCGCUAACAACAUCAAAUUGUGAAAAUUAUG
AAAGAAACAUGAUGACAAGUAGUAAUAUAAACCAUGGAAUUGCUAGUUCAGAUGAUGAU
GAUGGAGGCAAUUGUGCAAACAGUAAUGAUAGCAAUUCAAAUAAAUUAUUAUUAUUACC
ACGUCGGCGUACUUUACCCAGGCGUAAACGUCAGCCAUCAGCUAAGAAGCUUGAAGCGAC
AGAGUCUUUUGCCGCAGCAGUUAAGUCUGAUCAUCCCUUACAACAUUUUUCGUAUCGUCA
AAGUCAAUUAUCAGCAACGAUGUCAUUGGACUCCACAUCCGGGAGUGUCCCACUAUCAUC
GCAAAGUACUCAGUAUAAGCAUGGUAAUGUACUAUGUAGUGUCCGACUAUAUGUGAUGC
CUGACCAGACAGCUGCUGGUCGUCUUGGUGGUGUUCAUGGUGAAGAUGAAGUGUUAGAA
AUUAACACUUGGGAUACUAACGAUACAGAAGAUGGUUGGCCGAAUAACACAUUCAUGAA
UGAAAUAGGUUCUAGUGGUUACAGCGGUGGAGUUGAUGAUUAUGAUUAUGAUAAUGGAA

AUCGUUCAUCUAGAAAACUACCAUCUGGUUGUUCAGGAUUAGUAUUACGAGUCGAAGAU
UUUGUUGAAUGGGUUCGUGGUGGACUGAUGGACGAUGAGAAUGAUGAUUUUGAGUCUAG
CCAUGAGUCAUCAGAAUCCGAAUGUGUUGAUAACAGUUUAAAUGAUCAAAAAAUCACCA
AAACUGAACUACCAGAGAAAAAGAAUGAACAUAAACCAAAUUCAUUACCAUUUACAACA
GACGUCAAGAUUGAAGUUAAACAAUUGCAAAAAUUAGGAAGUAAUAAUAAAAACAAAAU
AAAAUUAGAAUCAACUGUCACGGUAGAUGUGCUAAAAAAGAAAUCUCUGAUCACGGAUA
CUUUGAAAACAGAAAAUGAAUCACCAUUGACAAUCGUUGUAGAUAAAGUAAAAACAGAA
CAAAAAGAUGUUGAUCGUGGAAAAGACAAAACAGAGAAACGCCAUAACAAUUAUAAUAA
UUGUUACUCCAAGUCUGUUGUUGAGGAGGCACUAGAAGUAGAACAUGUACCUUUAGCAU
CUGCUUUAAUACAUAAAGUACACUGUGGUUGUGUUAGUCACCGGGGUCAACACUGUCGC
UUUAAAUUGGUUAAUGAUAGGGAUACUGGCACAACAAAACACAAUCAUCACCACUAUGG
AUUGAAAAACAGUUGUAUAAAACAACAAUUAGUUGUUAUGAGUUACUCGCGAUACUGUA
GGUACAGAGCACACUUACAACGAAGACGUAAUGAACGAUCAAAUGAUGAAGAUAUAAUG
AAUUCAACUCGCGUUCUUUUUUGUCGAGAUACAUAUGACUACCCAGCUGAACUACUUCUG
GAUCCUUUUAAUGCUCAAAAUUUUACUCAGAAAAAUACUGCCGUCUUAGUCAACCAUAU
GGCGCCCAAACUCAAAGGUUGGCCAGCUUAUCCGGCAUCAUGGAAGUCAUUUCGAUGGAA
CCGUGUACGUCGUCGAUUGCGUCACUUAGAACGUGGAAGUAAUGGUAAUAAUGAUCGAC
GUAGACGUCGCCUUCGCCAAAAACUCAAGAAGAAAUGUCAGCGUCAAAAUUUAGCAAGU
GCAGCAAUGGCAGAUAUGAGCUAUUCUUCAUCGUCAUCAGACGACAAUUCAGACUCUGA
UGGAACAUCAUCAACUACAAAUGAUGAUGUGACAAAUACCAUAACUACAACUACUACUU
CGGAUUCUGAUGGUGCAUCGCCAUCAGUGAUAACUAAAACCACUAAUAAGAAUCUCUCU
GCUGCUAUAAUGAAAGUGAAUGGUGGUGUUCAUUUCAACAAUAAAAACACUGUAUCCGC
AGAUACUAUUAACAAACGUCUGUCACUUAUACAACCUGCUCAACAGCAACCUACAAAACG
UCGUCGUGUUCACUCCUCUAAAAGUCCACAGCAAAAUCAACAAGAAGCGUAUCAACAUUC
ACAACAAUUCAAUAAAGAAGUACAACAGCAAAAAAUACAUUCAUUUGACCGACAAUUAU
CAACAUCUUUAUCUACAGUACAACAUCCAACUACUAAAAUUGUUCAUCAUUAUUUACCAC
CUUCAGUAUCUAUAAAGCCAGUUAUUGUAUCUGAUACUAACACAAGUAUUCAACAACAA
CGAUUUUAUGAAACUCCAUUGCAAAACAGGCAAAAACAACAUACAACUGUUGUAAGCUC
ACAGUGUCAAAAUAAUAAUCAACAACAAACAUUAUUUCCACCUGUCAUAAUUGAAUAUG
GUGAGGAUAAUAACGAUAAGAAACAAAAGUAUGGAUACCAAAGACGUGUAGGGUAUCAU
GAUUUUGAAGACAAUCAUAAACAACAACAAGAACAGGAAAAGCCGCGUCGGUGGUUAGU
UGAUGCACUUCGUCUUGGAGGCUUGGAAGUUACAGCUGUUCCCUUAAAUGUUAGUGGAA
AUAAUGGUAACUGUAAUGAUGGCAAGAACUUUGGUUCCACAGUGACUACUGAAACUUUA
AAAAAUCAAAAUCAGCUGCGGCAAAGACAUUGUGGAGUUUCAUUUGAAUCAUUAUCAUC
CACCAAGAAUGAACAAAGAUAUGGUAAUAAUGAUUCAAAUCUAGAUACGGAUUCUGAUU
CUGUGUUGAUGACAGUAACACCUGAUGUUGUUUGUAUAUUGAAUAAGUCUAGCCAACAA
CAACAGACACAAAGAUCGCAUUAUCAACAGACAUUGUCUGAUGGUGUUUCCAUUUCAAC
AGAAUCAGGGAUUUUGCAACAAUAUCGUCCAGUGAACAUAAAAAACAAAAAUAGUAAUC
CUGCAUUACAUGAAAAAAUAAAUCAAGCAAUCAAUUCUCUAAAACAACAACAGUUAAAC
ACAUCUACCAGUAACAGAAAUAACAAUGUAAUAGUGGUGGAUAAAACUAAUUACCAAUA
UAAUAAUAGUAAUAGUAAACGACCUAAAAAUGAUGAAAUUUGGCAAGAAGCUUGCCCAC
CGGCGUUAUUAGACCUUACUGUCAAACCAUCGCCAAACACUAUAAGACGACAAAAGAGAC
CUACUGUGAACAAAAAGGUACAGGCCAACCAAAAAAUUACAUCACCAUUCCACUCUACUG
CCACAAUGAGGUCUGCUGCAACUAAUUUAGAAAUUACUAUUGUACCAGUUUCUGCAUCA

ACUACUGGUAAUCCAAUAAACCAUGCAAUCAACAAUGUAAACGGCAUGCAACGACCACAA
AAACUCAACUCACACAAUAAUUUGAUUGAUGGUUCUGAACCACCGUCCACGGUCUGUUCA
UCAUCUUCUGUGGAACGCCAGAAUGCAGUGUCUCCAUUUAUUGUAAAUAUGCUUUGCAG
UGACAAAAAUCAACAAAAUCGACAGCUUAGUUGUAGUAGUUCAACUACUAAUUCUUCAA
AAAAACAAAUACUAAUAAUGACAACCUCUUCUCCAUUAGCUACAACAGAUAGCCAACAAA
ACAGUAAACAUAAGUCACAGCAAAAUAAAAAAAAAAACUAA

>F01_transcript_2032

AUGUCCACGCGAGACCGUUCGCCGUCGCCGCCGCCGCUGCUGUUGUUGACGGCCAUG
GUCAUCCUGUCCGCCGUCGUAACAGGUAAAAACAAUUUGUUGAUUUGGUUUGUCGCCGU
UAAACGUGCCGUACGUAUUAUAACCGGUCUCCGUCAGCAUGUGUCGCGACGAAAGGUUU
UUUUUUUCAAUUCCGUUUUAUUUUACCGGGAGACUCCUAUUACGAGUGCGAUUAUUU
AUAACCGCGCACCGGUAUACGCGCGACGCGAACGAAACGUGA

>F01_transcript_11560

AUGAAUUUAAUGAAGGCAGAACGAAUAUUCACAACGAUCUUCCAUUUUGAGGUGACCAU
GUUUUGGGACCAAAAAGGGAUUAUUCUGGUCGAAUUUUUGCCUCGGGGGGAGAUGAUUA
AUGCGAAACGAUACUGUGACCAUAAAAAAGGUCAGGAGAGCAAUUCAAACCAAUCGGAG
GGGAAUGUUGACAAAGGGAGUGUGAUUGUUGCACGACAAUGCUCGCCCGCACACGGCGA
ACGCCACAAAAGUGCUUUUGGACUCAUUCGGUUGGGACAUUUUGAAGUACCCUGCAUAU
UCCCCUAA

>F01_transcript_11978

AUGAGUUUAAUGAAGGAUGAACGAAUAUUCACAACGAUCUUCCAUUUUGAGGUGACCAU
GUUUUGGGACCAAAAAGGGAUUAUUCUGGUCGAAUUUUUGCCUCGGGGGGAGAUGAUUA
AUGCGAAACGAUACUGUGACCAUAAAAAAGGUCAGGAGAGCAAUUCAAACCAAUCGGAG
GGGAAUGUUGACAAAGGGAGUGUGAUUGUUGCACGACAAUGCUCGCCCGCACACGGCGA
ACGCCACAAAAGUGCUUUUGGACUCAUUCGGUUGGGACAUUUUGAAGUACCCUGCAUAU
UCCCCUAA

>F01_transcript_13207

AUGGAAUUUCGCCGCAGCAGCAACCAGGUGGAAAUGUUUGCGAUUGAACUGAUUUGCAU
UACCCUGAGCGUGGCGGUGUUUGGCAAAAUUGAUGCGAUUAAAGAUCUGGAUGAUUGCA
UUUAUCAGCUGGGCCGCUGGGGCGGCUUUACCAGCCGCAUGACCAAAAAAGAUGCGAUUU
AUAACGCGUAUUAUAACAUUAGCCAGAACAUUAACACCGCGAAAGCGGCGAACGCGGGCA
ACAAAAAACCGAUUGAAAAAGAAGGCACCCAUUUUGUGUAUGCGUAUCAUGAUGAUAUU
CUGAACCGCUUUGUGGAAAAAUAUGGCGAUACCCUGAACACCAUUUUUAACGAUCCGAU
GACCUGGUGCUGCAACAACAUUAACACCUUUAUUAAAGAUGAUGAACGCAACAACAAAC
AGAUUUGCGAAAGCAACACCAACGCGCUGCUGAACGAAUUUUUUAGCUUUUUUAACAAC
AGCCAGAUGGUGAUUCUGGUGUUUAGCUUUACCUAUCUGGAUAAAAAAAAAGAAAAACU
GAAACAGAACGAUAUUAUGAAAACCUAUUUUAACUAUAUUGGCGAUAUUAUGGUGCAGA
AAGGCUUUGGCACCACCAUUAAACGCCCGUAUGAAUAUUGCCAGUUUAUUCUGACCCCGC
CGGCGGUGCCGAGCACC

>F01_transcript_13633

AUGUUUUGGGACCAAAAAGGGAUUAUUCUGGUCGAAUUUUUGCCUCGGGGGGAGAUGAU
UAAUGCGAAACGAUACUGUGACCAUAAAAAAGGUCAGGAGAGCAAUUCAAACCAAUCGG
AGGGGAAUGUUGACAAAGGGAGUGUGAUUGUUGCACGACAAUGCUCGCCCGCACACGGC
GAACGCCACAAAAGUGCUUUUGGACUCAUUCGGUUGGGACAUCUUGAAGUACCCUGCAU
AUUCCCCUAA

>F01_transcript_19713

AUGGAUUUUUGCGGCUAUCUGUUUGCGAGCCAGACCUAUAGCCAGCUGAAAAUGAGCGU
GUUUGAUCCGAAAAGCCGCCAUCUGCGCGAAAUUCUGCUGUUUUUUUUUAACAGCAAAA
AAAGCGCGGCGGAAGCGCAUCGCAUGCUGAGCGAUACCUAUGGCGAAGCGGUGGUGAGC
GAACGCACCUGCCGCGAAUGGUUUCAGCGCUUUAAAAACGGCGAUUAUGAUGUGGAAGA
UCGCCAUGGCGGCGGCCGCAAAAAAAUUUUUGAAGAUGCGGAACUGGAAGCGCUGCUGG
AUCAGGAUGCGUUUCAGACCCAGGAAGAACUGGCGGAAAGCCUGCAGGUGACCCAGCAGG
CGGUGAGCAAACGCCUGCAGGCGAUGGGCAUGAUUCAGAAACAGGGCAACUGGGUGCCG
UAUGAU
CUGAAACCGCGCGAUGUGGAACGCCGCAUUCUGUGCAUGUAA

>F01_transcript_21226

AUGUACACCGCGGAAAUGUUCCCGACGGAAAUUCGACAUUCUUUACUGGGCAUAUGCUCC
AUGUUCGGCCGAAUCGGUUCGAUGGUGGCUCCGCAGACGCCCUUAUUGGCGACUUAUUUC
GGUGAAUCGGCGCCGUUACUACUAUUCGGCGGAGCUGCUUUUCUAUCUGGUUUCUUGGC
GCUCCUGUUCCCAGAAACGUUCAAUAAAAAAAUGCCGGACACGGUUUUGGAAGCUGAAG
CUAUCGGCAAAACUUCUAGUUGA

>F01_transcript_22242

AUGAAAUGUAUCCUUCUCCACGACCCUCGUAAUAACGUAUUUUCUAACCUGGAUUUUCGA
AAUCUUCUUUUACUUUUAAAAAUUCCUUCGUCUUCUACGGUAUCAUCCCAUAAAUUUUC
AUUUUCGUUGUUUGUUGAUGACCGCAACGAUGUAACCUGUGUUAUAGGCAGCGUAGAAG
UUCUUGGUACACUUGAAGAAUAA

>F01_transcript_25009

AUGUUAUACCCAAGAGCGGUCGGGUUAAUGGAAAAAGCACGAAACGUGGUCAACGUGGC
GACUGAACGCGGCGUCGACGGUCGUACGGAUUCUCGUCGUCGAAAUGGCUGGAUCGUCGU
CGAGUCCCGGGCGAAACUUUUGCCGCCAAAACGAUUAUUCAAAAAUCAUGCGUCGGCGGU
AGGACACGAGUUCGGUUUAUAA

>F01_transcript_25108

AUGGGUGUGUGUAGGAAAGGGUUAGAACAAAAGAACAAAAGAAAAAAAGAAAUAGAAAU
AGAAAGUAGACUUUUAUUGAAUAUAAUUACGCGAGCAUCAAUUUUACGUUCGGCUAUGG
CGGCUACUCUCGCACGACAAACCUCCGUACGCGCGGCACACGCACUCCCGACAUCGUCGU
CGUUAUCGCGAUAA

>F01_transcript_25501

AUGAAAUCGUCUCCAGUUAAACGCUUUUUAGUUCAAUUAACUCUGUCAGUUGUAUUUUU
UGAUAACACCAUAGCAAAACCACAUGAAGAUUGGUGUGAAAUCCAUAUUGUUAAUAAUG
ACAUAUUAUUAAAGAAUAAUGCCCGUGACUUAUGCAAAUAUUUCUUAAUUUAUUUCUU
GCUGCAGACGAAGAAAACCAGAAUUCUUUAUUUGAAGCCCAGCAAACAUCCUUACAAAA
AGCUAUAGAAGACUUGCACUAUGAUAGUUUAACCUACAUUGCGGCUAAAACAGGCUAUG
AUGCAGCUACAAAAGGAUACGAUGCAGAAUUUUCACAGACAAACCUACAAUAA

>F01_transcript_25739

AUGAUUACUAUUUUGAACUAUAUUUUACGUAACUUAUGUUUCGACAAACGUUUUAUAGA
ACGGUAUAAGACCACUCCAAUAUUAAUCACUGUUCGUUGUAUUGAACUCAAUAAUUCAA
UUAAUUUUGUUUUUUUCACAUUUAUACUACAUUUCCAAUGUAUAAUCAUGAAUGUUUGA

>F01_transcript_27269

AUGUUUAAAUUUGGUGGAACUGUCAAUGCCCACUGUAACCACCACCAAUAUAAUAAUAU
AUAUUUUAUUAGAAAAAAAUUAAUUAUAAAUAUUAUUAUUUUUGACCAUAUAUGUAUAU
CUUCUAUAUCGAUGGUAACCAGAAACCACCCUUGUUUUAAAGUCUAUAAGUACAACCAG
UAA

>F01_transcript_27274

AUGGUUAUUUAUUUUAGUAAACGACGUAGACGUGAUAUGAUGUAUACUAUAUUUUUCAA
ACAUUAUUUAAUUCAAAUUCGUAAAAUUCAACCUUUUCUUUUCGACAAAUCAUCUAUAA
AAAAAAAUCAUAACGAAAACGUUUGUUCGAAUAACGACAAAUUUCUUCCACUGUUCGUU
AAAUUGAACUUAGAUUGCCGGAUCGUUCGGAAGAAAACGUUCGGAAUAUACCGAACAUG
A

>F01_transcript_36698

AUGGGCACAAUCGAAAAGGUUUUCAUGUUUUUAAAUACACCAAAGAGGCAAGCAGAAUU
UUCAAAAUAUAUUAUGGAACUGAAUCAAUCACAAACUCGUAUCGAAAAACUAAUACGAU
UGUGUUCGACAAGAUGGGUUGAAAGGCACAACUCGGUAGAAGUUUUUGAACAACUUCUU
CCAACUGUAUAUUCGUGUUUAGAAGAAAUGACUAGUUGGAAAGACAACGAAACAUCGGC
UAAAGCUCAUAUUUUAUUAUUUUCUCUAAGAGAAUAUGAAUUUAAUGUUGGUCUUAUUG
UUCUAAAAAAUAUAUUUCAAUACACAGUCUCUUUAUGCAUUUACUUACAAAACCUUAAC
AUAGACCUUAUUCAAGUUCCUAGUACAUUGAUAAUGUUGUACGUCAAAUACAAUUAG

>F01_transcript_37760

AUGGCUGUAAUUUCCCGUCUAAUGUUUUCUUUUAACUCUAGUAGUAUUUGUGGCUUGUU
AGUGAAUACUUUAGAUUUUAAAAAACCCCACAAAAAGAAAUCAUGUGGAGUCAAAUCAG
GAGAACGUGGUGGCCAGGCAAUAUCAUCAUUGCGCGAAAUCACACAAUUCGAAAACAUU
UUUCUAAGAAUAGCCAUUAAAAUAUGCACUGUAUGGCUGGUAGCACCAUCUUGUUAG

>F01_transcript_37767

AUGAAUCGAACGAUAGCUUCUCCCGCCAAGUGCGAAGUUCGUUCAGUUAUUCGAUUUUU
GCACUUCAAAGGGACUUCGCCUAUUGAAAUUUAUCAGCAACUUACCUCUGCAUACGGCUC
AAACAUUAUGGACAUCAAGAACGUAAGGAAGUGGUGCAGGGAAUUUUCGGCGGGCCGAA
CAAACGUCCACGAUGAGCCGGGCAGGGGACGUCCAUCCAUUUCCGACGAGACCGUUGCAC
GUGUGGGAGCACAUUUGCGCGAAGAUCGGCGUUUGACGGUUAGGGAAAUUUAUGAAAUG
GUUUCAGAUGUUUCUAAAACCACUAUUAAUAAGAUUUUAACAGAAAAUUUGAGAUACCA
UAAGAUCUGCGCCCGAUGGGUACCUCGAAUGCUGACCGACGAACACAAACGAAAGCGUGU
UGAUUCGUCUCGUGAAUUUCUGCGCCGAUACGAGGAGGAGGGGAAGGAUUUCUUGAUU
CUAUUGUGACUGGAGAUGAGACAUGGGUCCACUAUUUCACCCCCAAGACAAAAGAACAA
UCCAAGCAGUGGCGUCACCCAAACUCGCCGAGAGUCAAGAAGUUCAAAGAGACCACCUCA
GCCGGGAAAAUCAUGGCUACAGUCUUUUGGGACCGACAUGGGGUAUUGCUGGUCGAAUA
CCAACCUCGAGGAUAA

>F01_transcript_39455

AUGAAAAAUAAUUUACUUUACUUUAUAUUUUAUAUUGUACAUGUACGACAAACAACACU
UCCCGAGAACGGCAAAAAUCCAUCCAUAAAGUCGGAGAACCCUACCACAACUGAUUUCGU
UGCGACCGGCCACCGCCGGCAGUACUUAUCAUUAUCGCAACGGACACGAUAA

>F01_transcript_41799

AUGCUGAUCGUAUUUUUCAGCAUUAAAGGUGUAAUAAUGACUGAAUGGGUACUCCAAGG
ACAGACUGUUAAUCAACAUUAUUAUCUACAAGUCUUGACAACACUCGGAGAACGAUUAA
GAAGAAAGCGGCCUGAAUUGUGGGAACACGACUCUUGGGUCUUGCAUCAAGACUUAUCU
GUUAAGCAGUUUCUGGCCAAAAAUCGAACACCAGUGUUACAACACCCCCCUUGUUUGCCA
AAUCCUGCUCCAUGUGACUUUUAG

>F01_transcript_44077

AUGGAAAGGGACACCACGAUUAAUUCAACAGUGUACUGUGAGACAUUGAAGAAACUGAG
GCGAGCGAUCCAGAACAAAAGGCGGGGAAUGUUGACUGCUGGCGUUCUUUUCAUUCAUG
ACAAAGCGCGUCCUCACACCGCAGCACGAACUCGUGAAGUUUUGGAUCAAUUUGGUUGGG
AUGUUUUUGACCAUCCACUCUACAGUCCUGAUUUGGCCCCAUGUGAUUUUCACUUGUUCC
CCAAUAUGAAGAAAUGGCUUGGGGCGCAGCGCUUCGUGAACGAUGACGAUUUGAAAGGU
CGCGUCAAUGAGUGGCUAAAUGAACAGACGGCAGAAUUUUAUUCAGUGAGAAUUGAAAA
ACUUCUGUAUAGAUACGAUAAGUGCUUAAAUGUUGGUAGUAAUUAUAUAGAAAAAUAG

>F01_transcript_44187

AUGGAGGAUGAAACAUGGGCUCAUCACUAUACACCCGAAACGAAGCAGCAGUCCAAACAA
UGGCGCCACCCGACGUCUCCAAAACCGAGAAAGUUCAAACAAACACUGUCAGCCGGGAAG
GUGAUGGCAACAGUGUUUUGGGAUCGAAAGGGAGUAUUGUUAGUGGAUUUCAUGCCUCG
UGGAACCAAUAUCAAUGCGCAAAGAUACUGCGAAACAUUAGAAAAGCUUUGUUGCGCCA
UCAAGAACAAACGUCCAGGAAUGCUGACAAAGGGGGUCUCAUUUCAUCACGACAACGCGU
GCCCGCAUACGACCAAUUAA

>F01_transcript_45682

AUGAGAGUAUAUGAGUUGUCUACUGUAGUAUUUUCUGUGCCUGGAACACAAUUCAGUGU
AGAGAGACUUUUUUCUGGUCUUAAAUUCAUUUUAUCACCUUACAGAAGUAUUACUAAUG
CACAUAAUUUGGAGGAUCAAUUAAUUGUGCAGAUAAAUAGGAAGUUUUGA

>F01_transcript_46872

AUGUUCGCAAUUAAAUUGAUCUGUAUUGCUUUAGCUGUAGGCGUUUUUGGCGAAAUCGG
UGAUAUGACACGUUUUAAUUAUUGCAAAUAUCAACUUGGUAGAUGGGGAGAUUUUACAU
UAAAUAUGACUAAAACAGAAACCAUCCAUCAUGCGUACUUCAAUAUACGUCAAGAUAUU
AAUAUUAAAAAAGCUGCUAUUUCCGAAGCAGAUGAAAAAAAGACUAAUGAAAAAAAAGG
UAUACUAUUCAUGUAUGCCUAUCACGAUGACAUUCUGAAUAAAUUCGUUGAGAAAUAUG
AACACUCUGUGAAUACCAUGUUUUAUGAUCCAAUGACAUGGUGUUGUAACAUAAUAGAC
AUGUUUAUAAAAGACGAUGAAAGAACUAAAAAAGAAAUUUGCAAAUCAAAAAUACAAAC
UAAUCUAAAGGAAUUUUUCAUCAAAUGGUCAUACUGGUAUAUGCAUUUACGUACAUGGU
UCCUCCAGUCGCUUAAGGAAAAUGCAGUCCAACGCUUUGAUAAAAGUAUUGAAGUAUUA
AAACGAAAUAAUAUAAUGGAAGCGUAUUUUAAUUGCAUUGGAAAUUUCGUCAUGCAAAA
UGUUGCUGUAACCAGUGUAAUGAGCCCACAAAUAUAUUGCGAAAGUUAUAGCAAUUCAC
UGUUAAACGUAUCGGAAGUACCUUCAACAUAA

>F01_transcript_47954

AUGACGUCGCCGCGCCAAAAGAAAGCUCGAAUGAACAAGUCUCGAGUGAAGUCGAUGGC
AUUGUUUUCUUUGAUGUCAAGGGAAUUGUACACCACGAGUUUGUACCCCCUGGCCAGAC
UGUCAAUGCUAAAUUUUAUAAGGAAGUGCUUCAAAGGCUGAAUCACAGAGUCACCCGCG
UCCGAAAAGAGAUCCGAGCGUCAUGGAAACUGCAUCAUGACAAUGCGCCAUCUCACACUG
CCUUUGUUGUUACCUCCUACUUGAUUCGGCUUGGUGUCGAAACGAUUCCCCAGCCACCCU
CACGUUGGCAAAAAUGUAUUGAAGCGAAAGGGUCUUAUUUUGAAGAAUAUUAA

>F01_transcript_48017

AUGAUUACCAAGUCUUUGUUUUGCACGAACGUGAGAAACGAAAACGCCUAUUCUGUGUG
CACUGUGCUGCACGGUAACGAACAUGUGACGACGGCCCGCGAAUCGGUUACCGGCCAUCA
CGUUAAAAACGUAUUUGAUGGAAAAUGGAAAACGCGCGUUUUCGUGUAA

>F01_transcript_49314

AUGAGUACUACAAGUAUGGAAAGACAUUUGAAAUCAACAGUAUUAAAAAUAAUUAAAUU
AUUACACGGGGAUUAUGGAGUUGGAUCUAUCCAGACAUGCUUUAAAGUUCGUUAUUGUA
AUUCAACAACAAAAAUUAUCAUUUUUAAAACUAGACAUGGUCCACAUUUAUUUCUUUCU
UCUGUAAUACCAUUUAUAAAUAAGCUCCAAGAAAAACCAAUUCAAUUAUCAUCACUUUA
UACUGGUGCUUGUUUGUUUCAAUGUUACAAGUUUAUUCAGAAUUAUCAGAAAGACCUCA
UUAGAAAGUUAGCUGAAACAAUAAGUGAAACAACUAAACAAAAAAUGUUAAAUGUCAGU
GGUAUAUUAAAAUUUAAUUAA

>F01_transcript_50121

AUGAGUUUAAUGAAGGAUGAACGAAUAUUCACAACGAUCUUCCAUUUUGAGGUGACCAU
GUUUUGGGACCAAAAAGGGAUUAUUCUGGUCGAAUUUUUGCCUCGGGGGGAGAUGAUUA
AUGCGAAACGAUACUGUGACCAUAAAAAAGGUCAGGAGAGCAAUUCAAACCAAUCGGAG
GGGAAUGUUGACAAAGGGAGUGUGAUUGUUGCACGACAAUGCUCGCCCGCACACGGCGA
ACGCCACAAAAGUGCUUUUGGACUCAUUCGGUUGGGACAUUUUGAAGUACCCUGCAUAU
UCCCCUAA

>F01_transcript_53295

AUGAGUUUAAUGAAGGAUGAACGAAUAUUCACAACGAUCUUCCAUUUUGAGGUGACCAU
GUUUUGGGACCAAAAAGGGAUUAUUCUGGUCGAAUUUUUGCCUCGGGGGGAGAUGAUUA
AUGCGAAACGAUACUGUGACCAUAAAAAAGGUCAGGAGAGCAAUUCAAACCAAUCGGAG
GGGAAUGUUGACAAAGGGAGUGUGAUUGUUGCACGACAAUGCUCGCCCGCACACGGCGA
ACGCCACAAAAGUGCUUUUGGACUCAUUCGGUUGGGACAUUUUGAAGUACCCUGCAUAU
UCCCCUAA

>F01_transcript_53748

AUGGUUGAUUUUGAUACAUUGAAAGUCUCAGCAAGGCAUUGUAAAGUUGUCGAUGUAUU
CCGCUCAAUUUCUGCUCUAAGUUCGUCGUCAUCAACACUGCUACCUUGUGGACCACGGGG
UUCAUUCUCCAAGCCAAAAUUGCCUUCUCGGAAAUUGGCAAACCAUCGUUGCACUGUACG
UUCGUUGCAAGUACCUAGUCCUACAACCUGA

>F01_transcript_53844

AUGGUGAAUACAUUGCCUUCUGAUAUCUUUAAGAUGUCAGCUAUCUCACGUAACUUCAA
UUUACAAUCAGCCAAAGUUAUUUUGUGGACUUUCUUCAUGUUUUUCUGGAACAAACUGC
UAAAUUUGGGCGAUUCCAGAGCGUUCAAGCAUCAUCGAUCGAUUGAUCGAACGGCCGCA
UUUAAAAUCAGCGAACUAUUUCUCUACUAUUUGUCUCGAUAG

>F01_transcript_54417

AUGUUCGUGGCGGUCGGCUUCCACCGGGUGAAGACCGUCGGCGAACGGGCCACCAGGCAA
CGGGCGCCCGUGCUCGCCGUCGCGCCGCACUCGUCAUUCUACGACGGGCUCGCGGCCGUC
AUCGCGGGCGGUCCGACCGUCGUGGCCAAGGAAGAGAAUUCGUCCAUACCGGUCGUCGGA
AGUGAGGAGACGCGCGCACGCGUUUUUUCGCACGCGUUCAGACGGGCCGACGGGACCGGG
ACGCGAUCUCGUCGGCAAGGCGGCACCGGUCGCGGUUUUCGCGAUGUUUCGCGCCGCCGG
UCAGUUUCACACGUCGAACGCGCCCGUGCCCGUCGGCUCGGCGUAAUUGCUUUGUAG

>F01_transcript_57017

AUGUUGACGAAAGGAGUGUGUUUGUUGCACGAUAAUGCUCGCCUGCACACAGCCAUCACC
ACCAAGACGUGUUUGAACUUAUUUGGUUGGGAUAUUUUGAACAACUUUGCAUACUUCCC
UGACUUAAUGCCUUCAGAUUUUCAUUUUUUCACCUUCCUGGUGAAACAAUGGUGA

>F01_transcript_58521

AUGAUUUUAAAAUCUUAUAUUUGAAAACACAAAUUUACUAUCGUAUUCUGGACAUAAU
UAUUGUACAACUUAAACACCGUUUUAAUGGUAUGAACCAAGUAAUAAAACUUUUUACUU
CAAUAAUACCAAUUAAUAUAUGUUCAAUGAAUGAAAAUGUACUUAAACAUUCAACUACC
UUUUUAGUAAAUAAUUAUGAAAAUGAUUUAACAAUAUAUUUGGUCAAUCGAAUUAUUCA
ACUUAAACGUUCGUUUGAAAAUCAAAUUUCAAAAUUAAAUUCUAUAGAUGACUUGGCAA
CAUUUUUAAUUGUAGACAAUUAUUUAAUAGCGGCAAAUUUUCCAGAUUUAUGUACAACU
UGUUUUCUUUUUUUAACAAUACCAGUGACGGUGGCUAGUGCAGAAAGACGUUUUUCUAA
ACUUAAAAUAAUUAAAAACUGUCUUCGAAGUACGAUGUCCCAAAUCCGUUUGUCAAGUU
UAGCUAUAAUAUCAAUAGAAAAGAAAAUUGCCAAAGAAAUUAAUGCAUCAGAUAUUAUU
UCUACUCUAGCAAAUAAGAAGUCCAGAAAAAUGUUUUAA

>F01_transcript_58610

AUGAUGAGCAAGCUAAUGGAAAAAGAAAUUCAACUAAUUAAGUUAAAACGAAAAAGAAG
ACAUGAAUCUGAUUCGGUUAAAACAAUGAUUUUUUUUAUUCUUUAUUAUGAAAAUACUA
CAAUUUUAAUGAUACUUAUACUUAAUUACAGGCUCUCUUCAAAAUAUGCAUGUAGAGUU
AAAUAG

>F01_transcript_60213

AUGGCACCUCUAGGCAAGAAUUCAACCAAAAGAGGUUCCUUCCGGUCCCAAAAAGUUGAU
GACAUGAUUUUUGGAGUUAAAAAACUUGUUUUGACUUUUUUCGAGAAAGGAGAGUGGGU
GUGGCGCCACUGCAUGGACUGUCUCUUUGUCUCUGAGGUGUUGUGA

>F01_transcript_62589

AUGAUGGCUCCCACUGAAUCUUUGUUAAUACCCACUUCUGCUAUUAAUCGCAGAGUCAAU
CUUUUGCUAGCAACACUCGCACGCGUUCAAUGUUUUCCGCUGUUCUGCUUGUGGAUGGCC
UGCCCGAACGUGGUUCGUCCUCCACCGAUUCCUUGCCUGCACGAAAGCGUGCAAACCAAU
CAUACACACAUUUUAAGCUCACAACAUCCUGCCCAUACACUUGCACCAACAUUUCAUGAG
UUUCCGUCGCCGUUUUACCCAAUUUGA

>F01_transcript_62734

AUGCGGACGGGCGCGUGCACGCGAUCGUCUUUCCGGCCCCGUCCCGACGAACUUUUAUUC
GGAGGAAAUCCUCCGGCACGGCCGGUGCGCACGGUGUUGAGUUGGUUGGAACGUGCGCGC
GCGCGCGCACGACGACAGAUCGACAUGCCGUAG

>F01_transcript_64025

AUGUUUAGUCAUACUGAUAAUAAUAUGAAAAAAAUUAAUUUUUAUACUAAACUUGAAAU
UUUUUUAUUUAAAAAAAAAUUCGAAAAAAAGGAACUUUGUUUGAAAUUGGACUUACUUG
GUAGUUGUUUGAAUCAUUUCUUCAAAUUUAAAAAAAAACAUUACAUUUUUUAA

>F01_transcript_65818

AUGAUAAAUGGCACAUUGCAUCCCACAAUUAACUUCCGUCCAAAUCGACGUCUGACACUC
CCUAUGUCCACGCCGUCUUUUGAUUUAUCGUUACCGUCUUUUGUUGAGAGAUUAGAAGG
AAGAACUGAUGUAGCACUCAGGGAAACUGAUUUCCCAACUCUGUUUAUGUAA

>F01_transcript_65937

AUGGAUUUUAAAGAUAAAAAAAUGCGUAGAGCUCACGGAGGAAAUUAUUAUGAACCACU
ACCUUCAACUAGUUCAUAUGGUGGCGAAUUCAUUGAAACUGAAAAUGAUGAUUUAACAG
AACAGCUAAAGGACAAAGUGAAUACUUUGAAGUCUCUCUCAAUUGACAUUGGCGCAGAA
GUAAAAUACCAGGAUCGAUUAUUAAGAAAUAUGGAUCAUGAUUUUGAAACGACUGGAGG
AUUUUUAAGUAACACGCUUGGUCGAGUUACAAGACUUAAUAGAAAUAGUGGUGGCUACA
AUAUGUUGUACCUAAUUUUAUUCAGUGUAGCUGUCUUUUUUAUUUUAUAUGUAUUUUUA
AAACUAAGAUAG

>F01_transcript_67101

AUGUUUUCCCAAAUACAUCGAUUUUUCGGACAGCGCGUGGAAAACGUUAAAACCCUGUG
GAUAUUCAUAAACGUUUUUUCGUGGCUUACAGGAACGAAACUAUUGACGUUAGUUCCGC
UCGGGGUUCCAACACGGGUUAAAGGUAACGAAGUCGGAAAGGCCAUUAUUUCCGAUAAA
GCCGGGAAAGUUAUGUUCACUGUUUGUCGGGACUCAAAAGGUGUGAUGCACACUGAAUA
UUUGGAGAAACGAACGACAAUAGAUUCUGUAAGCUACCAAGAAACUCUAAAAAGACUUA
AAUGA

>F01_transcript_71490

AUGUUUUGGGACCAAAAAGGGAUUAUUCUGGUCGAAUUUUUGCCUCGGGGGGAGAUGAU
UAAUGCGAAACGAUACUGUGACCAUAAAAAAGGUCAGGAGAGCAAUUCAAACCAAUCGG
AGGGGAAUGUUGACAAAGGGAGUGUGAUUGUUGCACGACAAUGCUCGCCCGCACACGGC
GAACGCCACAAAAGUGCUUUUGGACUCAUUCGGUUGGGACUUUUUGAAGUACCCUGCAU
AUUCCCCUAA

>F01_transcript_71493

AUGUUUUGGGACCAAAAAGGGAUUAUUCUGGUCGAAUUUUUGCCUCGGGGGGAGAUGAU
UAAUGCGAAACGAUACUGUGACCAUAAAAAAGGUCAGGAGAGCAAUUCAAACCAAUCGG
AGGGGAAUGUUGACAAAGGGAGUGUGAUUGUUGCACGACAAUGCUCGCCCGCACACGGC
GAACGCCACAAAAGUGCUUUUGGACUCAUUCGGUUGGGACAUCUUGAAGUACCCUGCAU
AUUCCCCUAA

>F01_transcript_71708

AUGGUUAAAAUUAUUGAACAAGAUGCAUUGAUAGACAUUUUAUCUGAAGUUUAUAAAUU
AUUUAUUCUUAUUUUAACCAUACCAUCAAUUAAUGUAUCAAAUGAAAGAAGUUUCUCUU
GCCUCAAAAGAACCCUGAAAUAUUUAUCAAAAAUCAAUAUGUAA

>F01_transcript_72945

AUGAAUUUAAUGAAGGCAGAACGAAUAUUCACAACGAUCUUCCAUUUUGAGGUGACCAU
GUUUUGGGACCAAAAAGGAUUAUUCUGGUCGAAUUUUUGCCUCGGGGGGAGAUGAUUA
AUGCGAAACGAUACUGUGACCAUAAAAAAGGUCAGGAGAGCAAUUCAAACCAAUCGGAG
GGGAAUGUUGACAAAGGGAGUGUGAUUGUUGCACGACAAUGCUCGCCCGCACACGGCGA
ACGCCACAAAAGUGCUUUUGGACUCAUUCGGUUGGGACAUUUUGAAGUACCCUGCAUAU
UCCCCUAA

>F01_transcript_73025

AUGCGUGAACGUGCUAUUUUUGCUGCAAAAAAUCUCGAUUUUGAUACAAUUAAUUUCAA
AAUUCAACAGUCAUUGUCUGGCAAUAAUAUGUCGUUUAAAUCGAUCAACAUUGUUGUGA
AUACCGACGAAGCAGUCAACUUUCCUGAUGAAUUCUUGAUUACACUGAAAUUACUGGGA
AUAUGGUAUUCAAAUUUAACCUAUUACACACAUUUUACAUUCUAUAUAUCGUUAUUGGA
AUUGAAUAAAAUGCAUGGCUUAAGAGAUCCGCGGAGUGAAUAA

>F01_transcript_73324

AUGAGCUCGGCAGCUGUCCGUCUUCGCGGACCACGUCACGGUUCGCGUCCGGAGAGUCAU
GACGUUCCGUUUGAACGGUUCGCAGUCCGUUACGUUUACCGUGCCGGGGGGGGGGGGU
CAAGUCCGUCCGUCCGGCGCUCGUCUUCGAAAGAUUUCGCAAAUCUACUUGCCGACGGUC
CGCCCCCCGGGCAACGGGACGCCGGCGAAUUUUCCGGGGCAACGGUAACUCGGACGACGG
GAACAGGCCGGCCGAACGGUUUUCGUGCACGCGCGUCACGAAAACGACAUGGACGUUUUG
UACGCGACUGAUGUGUCGCGCGUCUCUGUCCGCGUGUCCGAGACCCGUCCGAGGAAAGCG
UGUCCCCGGGUCCGGCAAGCGGGGCCUUCUCGUGGACCGGCCGGCCGUCUGGAAACAUCG
AGCCGAAGGACGUUGUAACUGUCGGCUGGGCGGCACGACCCGUAGAAAAUCGGAGUCCGU
UGACACGUGCCGUCGUGUACCAACGCGAUCGUUUGUUUCGCCGCGCCGUCCGUCGCGUCG
CCGGUCAUCUGCGGCGGGUACCGGUAGGCCUCCUCCGGAAUGUCCGUCGAUGCCGCGCGG
CACAAGCAAUUUUGAUGAUCCCGUCUACCGUCCCGAAAUGCUCGCCUUUCAUGGGUGUUU
UGAGGCGUCGAAACACAAAGAAGUCGGAGGGAGCUACGUCCGGGCCGUACGGGUGCCGG
GGGACCGUCGGCACCCCCGAUUUCACCGGGUAGUCGGAGACGAUAGAGGCGGUGUGGUUU
UGUCUCUUCCCGGCCUUCCAGAAAGGCCUUGUGCCACCGAAACACUUGCCGAUCUGAUAG
GGCAUCUUCUUUGUAAGCCGACUCGAUCGCAGGAACAGUCUCCGUCGCGGUCUUUCCGAG
AUUCACGGAAAAAACUUGCGCGCAACUCUUUGUCCUAA

>F01_transcript_73688

AUGUUGAUCAAGGAAGUGUGUUUCCUUAAAGACAAUGCCCGUUCUCAUGUUGCGCGUGU
CACUAUCGAACUUUUCAAGAAAUUUGGUUGGGACAUUUUGACUCAUCUUCCUUAUAUCC
CUGAUUUGGCACCGAUAAUAAUCGACCUUUACACAUGA

>F01_transcript_75433

AUGUUAUGUGAAGUUUAUGAUGAAGAAUGUUUAUCAAGAGCUCGCGUUUUUGAAUGGCA
CAAACGGUUUUGCAGUGGAAGAGAGGACGUCGAAGACGAUGAUCGUUUUGGACAUCCUA
CCACAUCUUCAACAAACGAAAACGUUGAAAAAAUCGACAAAAUUAUUCGGCAAGAUCGU
CGAUUAAGUGCUAGAGCUGUAGCAGAGAUGGUAAAUAUUGAUAGAGAAAGUGUUCGUAA
AAUUUUAGUUGAAAAUUUAAAUAUGAGAAAAGUGUGUGCUAAAAUGGUCCCUAAAAAUU

UGACGAUUGAUCAAAAAUUCAAUCGCAAAGAAAUUUGUUCUGACACCUUAAAAAUUAUC
AAAGAUGAUCCAUUUUUUAUAAAUAAUAUUAUUACGUGUGAUGAAACAUGGAUUUUUAC
AUAUGAUCCUGAAACUAAAAGGCAAUCCAUGCAUUGGAAGACACCAACUUACCCAAGAA
UGAAGAAAGCACGAAUGAGCAAGUCGAAAUUCAAAGCAGUGCUCAUUGUUUUCUUUGAC
AUCAAGGGAAUAAUUUUUAUUGAAUGGGUUCCUAGUGGGCAAACUGACAACCAAUAUUA
UUAUAAAGAGGUAUUAAUUAAAUUAAGAGAACGUGUUAGAAAAAAGCGACCAGAUCUGU
GGAAAAAUGGCUGGGUUCUUCACCAAGACAAUGCUCCAGCUCACAGUGCAUUUUCAAUUC
AACGUUUUUUAACUGAAAAAAAUAUUUCUAUACUUCAACAUCCCCCGUACUCGCCAGACC
UUGCUCCUUUUGACUUCUUCUUAUUCCCAAAAAUUAAUAGUUUAUUAAAAGGAACCCAU
UUUCAAACGGUUGAUGACGUAAAAAUGAAAACGGCAGAACUUCUAAAAGGGCUGACUGA
AAGCGAUUGGCAACAUUGUUUUCAAGAAUGGCAGCGACGUAUGCAACAGUGUGUUGAUG
CUGAGGGAAGGUACUUUGAAGAAGCAGCACUGAAGCUUAAUCGAAAUAUAGCUCGACAA
UAUGCAUAUUUUAAAAAACUUUUUCAAUUGUCCAAUGAUCCAGCUAGUGUAAUAUUAAA
AGAUACAUUUACUUAUGAAUAUUAUAGGUGGGCGGUGUCAACGUUAAUGUCUCGUCAAA
AUACAGUUCCCAGUUCCGAUAAUUCUUCCGAAACUAUUAGUGCACUUAUACCGCUUUGGG
ACAUGUUCAACCAUCACACCGGAAGAGUAAUAUUAUUCACACCUUAUGUUUUAAUCACG
AAAUAA

>F01_transcript_77292

AUGAAUUUAAUGAAGGCAGAACGAAUAUUCACAACGAUCUUCCAUUUUGAGGUGACCAU
GUUUUGGGACCAAAAAGGGAUUAUUCUGGUCGAAUUUUUGCCUCGGGGGGAGAUGAUUA
AUGCGAAACGAUACUGUGACCAUAAAAAAGGUCAGGAGAGCAAUUCAAACCAAUCGGAG
GGGAAUGUUGACAAAGGGAGUGUGAUUGUUGCACGACAAUGCUCGCCCGCACACGGCGA
ACGCCACAAAAGUGCUUUUGGACUCAUUCGGUUGGGACAUUUUGAAGUACCCUGCAUAU
UCCCCUAA

>F01_transcript_78222

AUGAGUUUAAUGAAGGAUGAACGAAUAUUCACAACGAUCUUCCAUUUUGAGGUGACCAU
GUUUUGGGACCAAAAAGGGAUUAUUCUGGUCGAAUUUUUGCCUCGGGGGGAGAUGAUUA
AUGCGAAACGAUACUGUGACCAUAAAAAAGGUCAGGAGAGCAAUUCAAACCAAUCGGAG
GGGAAUGUUGACAAAGGGAGUGUGAUUGUUGCACGACAAUGCUCGCCCGCACACGGCGA
ACGCCACAAAAGUGCUUUUGGACUCAUUCGGUUGGGACAUUUUGAAGUACCCUGCAUAU
UCCCCUAA

>F01_transcript_78466

GAAUUUCGCCGCAGCAGCAACCAGGUGGAAAUGUUUGCGAUUGAACUGAUUUGCAUUAC
CCUGAGCGUGGCGGUGUUUGGCAAAAUUGAUGCGAUUAAAGAUCUGGAUGAUUGCAUUU
AUCAGCUGGGCCGCUGGGGCGGCUUUACCAGCCGCAUGACCAAAAAAGAUGCGAUUUAUA
ACGCGUAUUAUAACAUUAGCCAGAACAUUAACACCGCGAAAGCGGCGAACGCGGGCAACA
AAAAACCGAUUGAAAAAGAAGGCACCCAUUUUGUGUAUGCGUAUCAUGAUGAUAUUCUG
AACCGCUUUGUGGAAAAAUAUGGCGAUACCCUGAACACCAUUUUUAACGAUCCGAUGACC
UGGUGCUGCAACAACAUUAACACCUUUAUUAAAGAUGAUGAACGCAACAACAAACAGAU
UUGCGAAAGCAACACCAACGCGCUGCUGAACGAAUUUUUUAGCUUUUUUAACAACAGCCA
GAUGGUGAUUCUGGUGUUUAGCUUUACCUAUCUGGAUAAAAAAAAAGAAAAACUGAAAC

AGAACGAUAUUAUGAAAACCUAUUUUAACUAUAUUGGCGAUAUUAUGGUGCAGAAAGGC
UGCAUGUAUCAUUAUCGCAAACGCAAAAAAUGGUAA
>F01_transcript_78676

AUGGCCAAGUCUUCCGAAACAAUCUCAUGGACAAUGGUUUUUGGCAAGUUCAAUAAUUC
CAACAUCAUACGAACACUCAUUCGUCGGUCCAUGUUAAACAAUUCGCGCACACAGGUGAG
GUAUUUGUCUGUUCGCGAUGUUGAUGGGCAUCCAGAACGGACCUCGUCGGUGACGUCUU
CCCGACCUUUCCCGAACGACUUGUGCCACUUUUUUGCCUGCGAAUAG

Mule transcripts

>F01_transcript_6705

AUGCCGAGAGGUGGUCGUCGUCAUCAUAGACGACCAAAACUUCCUCAGUCAAUGAUAGA
GUUUCAUACACAACUUUCCGGGCGUUUUAAUUAUCUGACUCUUUGUAAUGAUGUAUCAU
UGUAUUCGGGAAUGAUUACCAACAAUUCCGGUGAAGGAUGUACUUUACUUUUUAUUAUA
CCUGAAGUCAAGAAAAUAUUUAAAACUGGAUCGACAUUUUCGUUCAAUUGUAAUGUUGU
UCUUGGACCAUCAUUUGGGAAUGCAUUACAGAAAUUAUAUGUUAUUAUGGGCAUGCAUA
ACAAUGUAGCGUUUAUCAUUGGGUUUGCUUUAAUGUCCGAAAGUACGAAAAGUGCUUUC
AAAGCACUUUAUUAUAAGAUUCUAAGUAUUGAACCAAGAUGGAAACCCCAAACUAUAAU
CUGUUGUUUCAAUACAGAAGAAGCUAAUGCAAUUACAGAAGUGUUUCCAAAUACAAUUA
UUCAAGGAUGCUGGUUUUACUUUUGUAAAAAAGUGUGGGAACAAUUGGAAGGCUUAGAU
CUUCGUAGUUUUUGCAACACUGAUAGAAUAACGUUUGAUUAUAUGCAUAUGUGUACAGC
AAUACCUUUGUUACCAACUGAACAUACCGUAAAUGGUUUUAUCGACUUGAAAGCUAACU
UAAUACGCAUUUUGCAGCCAUACUUGCGCUCUGAAUAUUGUAGGAAAUUCAAUACAUUC
UUUACUAUGUUUAGGGAAUUAUGGUUUGAAGGAGUUAAUGGUGAAAUAAUACCAAUGAA
AGAUGCUCAGGUAUUACGUUCAAAUAUUGAGAUUGAUCUCUGCAGUCAACAAUUACAAG
CAAAUAUGGAACACCACACCAACCCUGCACCAUGGCCGUUUAUAAGAAGCCUUGUUGCUU
AUUCAAAGUUGGCCAUAUUUAAUUACCAGACGCCAAAUGAGAGUUAUUAUGAUAACUUA
GAGUAUCCACGCAGUACUUUGAUUAAACAUAUUGAAAUUUAUAAUCAGAAUUUGAUAGA
UUUUUAUGCAGGGCGGAUUCAUGUAUUUCAAUAUCUUGUGAAUGUAAUGAGUACAACUC
CAGGUUAUGUGUUUUGUGAUGAGUUGCAUAUACCUCUAGAAGAUGAUUCUUUACCCGCG
ACAUGGCAUUUGCAGAAAGUCGAAGCUUAUUAUGAGGAAAUUGAGAUUGAUAGUGAUUA
UAACUAUGUAUAUGAUGAUGAUGAUGAGUCUCUUUUGUUCGAUGAAAGCGAUACCUCAU
AUGAAGAUGACGAAUCUUCUGAUGAAGAGAUACCUAAUGAACCACCAUCACUUGUUCCU
UUCUUACCCAAUGAAAUGAAUUUUAAACCAGUAGUAGAAGAAAUGUGCUGUAUUUGUUU
AAGUGCAUGUCCACAAAUCAUGGCUUCACCUUGCAUGCACCAAUGUAUGUGUGUAUCUU
GUUACGAGUACUCCAUUAAAUUACGUCCAUUUCGGCACAGACCAUAUUACCGUUGCCCAU
UAUGUCGUAAUCAAAUUGAUUUGUACAUGAAUAUAAAUUAA

>F01_transcript_9097

AUGGAACUUAAUUGUGAGGAUUGUGAAAUGAAUUUUGCCACUCCCUAUACACUGAGAAG
GCAUUUGAAAAGAGUACAUAAUAUUUUAGAGGUAAUAACAAAACCAAAACAAUUGUCUA
AAUGUAUAUCCUGUACGAAUAACAUAACAUUCUCAAAAAGAAGUUUACUCAUCGAUCAU
UUGAAUACACAGCAUGGAAUGUCUAUUAAUGAAGAAAUGUUAAGCUUUUCAAAUUCAUC

AGAGUUUCGUAGUUGGCUAAAUGAAUAUGAAUUAGAACAUAAAUGUGAGUAUGUUCUGA
AAACCGGUAAAAAAAAGUGUGUCGGUGGUCAUAGAAGUUACUAUGAGUGUUGUCGAACU
GGUGUUUAUGAAAAACCACAUAACACAAAACGAAAAGUUAAGUCUCAAGGCAGCAAGAA
AAUGAAUAUUAACUGUACAAGUCAUAUUGUAUUAUUUGAACCCUUAAAUCAAAGUAAUU
GUACUGCAACUUUUUACAAACAACAUUAUGGUCACAAAGAAAAUGAACUUCAGCACAUC
AGUAUACCAAUUAUAAAAAAAAAUGAAAUUGUUGCCAAAUUGUCACAAGGCAUAAAAGU
GUCUGAACUGUUGCAUGAAAUUAAGGACAACAUUGGGCCCGAUUUAAAUAGAAUAGAUA
AAGUCACAAGAGGUGAUUUGCAUAACUUCAUAAAGAAGUAUAAUAUAAAAAUACAAAAA
GAGGAAGAGAACAUCACAAAUUUCAACAGCUGGAUAGAAGUAAUGAAAGAGAAAGGAAC
AAAAAACCCUGUUAUAUAUUAUAAAAAGCAAGGUGAAGUAGAUACAACCAAUAACUUGG
AUUCAACAGAUUUUUGCCUUAUAUUAAUGUACCCAGACCAAGUACGUAUGAUAAAAAAA
UUUGGACCUGGAAAACUAGUGUGUAUUGAUAAUAUUCACAGUGAUUCAAAUGAUCAUAA
UUUAAUAACUUUAAUGGUUGCUGAUGAUUUAUCAUUGGUAUUUCCAUGUUGUUUCAUGU
UCAUCAAUUCAAAUGAUACAAAAAUGUAUUCCAUAAUGUUUUCCAUUAUAAAAGAUAAU
GUUGGCGCCAUAAAUCCAGAAAUUAUUUUGACUAAUCAAGUGGAAGAAAUUUAUUCAGC
AUGGGAAAAUAUAAUGGGCCCAGUUCCUAAUACUGCUUUAUACUCAUGGUAUGUUGACA
GUGCUUGGAAGCAAAAUAUUUGCAAGAUCGUAGGCUAUAAACACCAAGAAAAACAAGAU
ACUGCAUAUAAAUCAUUAAAAAUUAUUCAGUCAUUGGAUGAUCAAAAUACAUUUCAUAU
GGCUUGGAAAGAAUUUUAUGAUAUUUUGAUUAAUGAUCCAGACACUCAAGACUUUGUCA
CAUACUUUUAUCAAACAUAUGGCAGUACAAUUCAGACAUGGGCAUAUUGCUACUGGAAA
GAUAAAAAAGUCAAUGUCCAUCUAGAAUCUAUACAUAGAACAAUUACACAUCAUUACUU
AAAGGGCUGUAAAACUGGACAGCUGGCAAAAAUUAUAACAACUUUAAGAAAAUUUACAC
ACAAUCAAAUAAUUAAAAUCACUAAAGAAAGACUAACAUUAAUCACAGAAAUUGAAAAA
ACACACAAGGAAAGUACUUUACUAAAUCAUACUAUUAUUAUUCAGGAUGAUGUUAAUAA
CUGGAGUAUUGAAAGUGAACACAUGUCGAAUCAAUUUUAUAAAAUAAGAAAAAUGAAUG
AUGAUAUAUGUUGUGCAAUGAAAUGUUCUGCUUGCAAAUUAUGUAUUCAUACAUUCCAU
UGCACCUGUCCUGAUUAUCAAAUUAAGUCCAAGAUAUGUCAACAUAUACAUAUGGUAGC
UCUGAAUUUGAUUUCAUUGGAAAAUGAUGUUGGAAAUACAUCAAUACCCUCAGAUAAUA
AUUUAGAUAUGACAUUGAAAGAUGUAUUUUUUGUUCAGUAUUUUUAUUUAUUUUAA

>F01_transcript_32208

AUGAGUACAACUCCAGGUUAUGUGUUUUGUGAUGAGUUGCAUAUACCUCUAGAAGAUGA
UUCUUUACCCGCGACAUGGCAUUUGCAGAAAGUCGAAGCUUAUUAUGAGGAAAUUGAGA
UUGAUAGUGAUUAUAACUAUGUAUAUGAUGAUGAUGAUGAGUCUCUUUUGUUCGAUGAA
AGCGAUACCUCAUAUGAAGAUGACGAAUCUUCUGAUGAAGAGAUACCUAAUGAACCACC
AUCACUUGUUCCUUUCUUACCCAAUGAAAUGAAUUUUAAACCAGUAGUAGAAGAAAUGU
GCUGUAUUUGUUUAAGUGCAUGUCCACAAAUCAUGGCUUCACCUUGCAUGCACCAAUGU
AUGUGUGUAUCUUGUUACGAGUACUCCAUUAAAUUACGUCCAUUUCGGCACAGACCAUA
UUACCGUUGCCCAUUAUGUCGUAAUCAAAUUGAUUUGUACAUGAAUAUAAAUUAA

>F01_transcript_76970

AUGCCGAGAGGUGGUCGUCGUCAUCAUAGACGACCAAAACUUCCUCAGUCAAUGAUAGA
GUUUCAUACACAACUUUCCGGGCGUUUUAAUUAUCUGACUCUUUGUAAUGAUGUAUCAU

UGUAUUCGGGAAUGAUUACCAACAAUUCCGGUGAAGGAUGUACUUUACUUUUUAUUAUA
CCUGAAGUCAAGAAAAUAUUUAAAACUGGAUCGACAUUUUCGUUCAAUUGUAAUGUUGU
UCUUGGACCAUCAUUUGGGAAUGCAUUACAGAAAUUAUAUGUUAUUAUGGGCAUGCAUA
ACAAUGUAGCGUUUAUCAUUGGGUUUGCUUUAAUGUCCGAAAGUACGAAAAGUGCUUUC
AAAGCACUUUAUUAUAAGAUUCUAAGUAUUGAACCAAGAUGGAAACCCCAAACUAUAAU
CUGUUGUUUCAAUACAGAAGAAGCUAAUGCAAUUACAGAAGUGUUUCCAAAUACAAUUA
UUCAAGGAUGCUGGUUUUACUUUUGUAAAAAAGUGUGGGAACAAUUGGAAGGCUUAGAU
CUUCGUAGUUUUUGCAACACUGAUAGAAUAACGUUUGAUUAUAUGCAUAUGUGUACAGC
AAUACCUUUGUUACCAACUGAACAUACCGUAAAUGGUUUUAUCGACUUGAAAGCUAACU
UAAUACGCAUUUUGCAGCCAUACUUGCGCUCUGAAUAUUGUAGGAAAUUCAAUACAUUC
UUUACUAUGUUUAGGGAAUUAUGGUUUGAAGGAGUUAAUGGUGAAAUAAUACCAAUGAA
AGAUGCUCAGGUAUUACGUUCAAAUAUUGAGAUUGAUCUCUGCAGUCAACAAUUACAAG
AAAAUAUGGAACACCACACCAACCCUGCACCAUGGCCGUUUAUAAGAAGCCUUGUUGCUU
AUUCAAAGUUGGCCAUAUUUAAUUACCAGACGCCAAAUGAGAGUUAUUAUGAUAACUUA
GAGUAUCCACGCAGUACUUUGAUUAAACAUAUUGAAAUUUAUAAUCAGAGUUUGAUAGA
UUUUUAUGCAGGGCGGAUUCAUGUAUUUCAAUAUCUUGUGAAUGUAAUGAGUACAACUC
CAGGUUAUUUGUUUUGUGAUGAGUUGCAUAUACCUCUAGAAGAUGAUUCUUUACCCGCG
ACAUGGCAUUUGCAGAAAGUCGAAGCUUAUUAUGAGGAAAUUGAGAUUGAUAGUGAUUA
UAACUAUGUAUAUGAUGAUGAUGAUGAGUCUCUUUUGUUCGAUGAAAGCGAUACCUCAU
AUGAAGAUGACGAAUCUUCUGAUGAAGAGAUACCUAAUGAACCACCAUCACUUGUUCCU
UUCUUACCCAAUGAAAUGAAUUUUAAACCAGUAGUAGAAGAAAUGUGCUGUAUUUGUUU
AAGUGCAUGUCCACAAAUCAUGGCUUCACCUUGCAUGCACCAAUGUAUGUGUGUAUCUU
GUUACGAGUACUCCAUUAAAUUACGUCCAUUUCGGCACAGACCAUAUUACCGUUGCCCAU
UAUGUCGUAAUCAAAUUGAUUUGUACAUGAAUAUAAAUUAA

PiggyBac transcripts

>F01_transcript_330

AUGUUUUUUCUUGUCGUGACGGUGCUGAGACAAUCCGUUUCACCGUUAUUGUUAUUCC
GAGCCGAGCAGUUCGAAUGGCAUUCGAACCGAUCGUCUCCGUCUGCUGUUGCGUUCAGCC
UGUCUCUCGUGGACGCACUUGGGUUUUGUUUUGUCGUGUGCUGCUGCAGUUUCCUGUCA
AACCAAUGUUUCCGUUUGUCGUGUCGUCUGUUUUGAAUCCAUCGACCGUGCGGUAA

>F01_transcript_562

AUGAAACCGUCCAAUUUAAAAGCUACUAUACAUACAUUCAAACAAACAUUCAAAAAGGC
AUUGCUAGCAAAUCAAGCUGUAUCGAAAUCAUGUACUAACAGCAAAUGUGUUCCAUAUU
CAGAUACACAUAAAUUAUAUUUUUGUGAACAAUUUCUCAAUAUACCAGUACUGAUCAUU
AAAAUAUUGUCAACCAUAUGUUAUGUUUCAACUACUUGUUGUUACCUGGUCAUCAAUUU
AAUCAAUGCUAGGUUGGUAGUUGAAAAAAUGUGGUAG

>F01_transcript_21843

AUGCAACCGCAAGAAGAUUUAUGCAUAGAUGAAUCGUUUGUAAAAUUUAUGGGUAGACA
CUGGUACUCAAGUGUUGAGUUAGCUGAACAGUUAAAAACCAGACAAACUCAUCUUGUAG

GAACUAUCAGAUCGAAUAGAAAGUCUAAUCCUAAAGAUGUAAUGCAAAAAAAGUUAAAA
AAAGGAGAAAUAGUAUCAAAGAGAAGUGAUACAAACGUUUUGAUUUUAAAAUGGAAGGA
UAAACGGGAUUUGUAUAUGAUAUCCACCAAACAUACCAGUGAAGUUGUUGAACAUGUUA
UAAGAGGUAAAAUUGUCAAGAAACCUAAGGUAGUAAUGGACUACAAUUCAGGAAAAACA
CCUAUUGAUCUUUCUGAUCAAAUGUGUUCUUACUCUAACCCUUUAAGGCGUAGUGCUAA
GUGGUAUAGAAAGAUAGCUCUAGAUGGACUUUUAAACAUAGCUGUUGUUAAUUCUCUAU
UAUUAUUCAACAGAAUUACAUCUUCAACAGUAUCAAUCACCGAUUUCCGUACUUCACUUG
UCGAACAAUUGAUCAAAAAAAAAAAACUAUUGA

>F01_transcript_21885

AUGCUGAUGAUGUCUGCCGACAUGAUGUUUGCGGUCGUUGGCGGCAGCGGACCGUGCUG
GCUACUGAUUUCUGUCGUGUGGUCGUUGCACGUGUUGGAAGACUCGGCCGCGGUCGCUGC
GUUACCACCUGUUAUACGGAUCGGUAUGUACUUUGGAAAAAUAAAUCAGUGA

>F01_transcript_25501

AUGAAAUCGUCUCCAGUUAAACGCUUUUUAGUUCAAUUAACUCUGUCAGUUGUAUUUUU
UGAUAACACCAUAGCAAAACCACAUGAAGAUUGGUGUGAAAUCCAUAUUGUUAAUAAUG
ACAUAUUAUUAAAGAAUAAUGCCCGUGACUUAUGCAAAUAUUUUCUUAAUUUAUUUCUU
GCUGCAGACGAAGAAAACCAGAAUUCUUUAUUUGAAGCCCAGCAAACAUCCUUACAAAA
AGCUAUAGAAGACUUGCACUAUGAUAGUUUAACCUACAUUGCGGCUAAAACAGGCUAUG
AUGCAGCUACAAAAGGAUACGAUGCAGAAUUUUCACAGACAAACCUACAAUAA

>F01_transcript_27806

AUGGUGAGCCAUAUUAGCGCGAGCACCGAUGGCGAUUUUGCGCCGAUUAGCUGCGGCCCG
GGCUGCCCGAGCAUUCAUACCAACGAUCGCCAGUGGCGCGAUGAUGCGAACGUGGUGGAU
GAUUUUAUGUUUAACCCGGAUAUUACCAACAGCGGCAUUAACCCGGAUCUGUUUGAUGU
G
CUGAUGCAUGGCAGCAGCCUGGAUUUUUAUAUGCUGAUUGUGGAUAACAAAAUUAUUAA
C
AACAUUGUGAUUGAAACCAACAAAUUUGCGACCCAGCAGAAAGAACCGAAACUGACCAGC
CCGUUUGCGCGCCUGAACAAAUGGUAUGAUACCGAUGUGACCGAAAUUAAACAGCUGUU
U
GGCCUGCUGAUUUGGACCGGCCUGGUGGCGUUUCCGAGCUAUGAACUGUAUUGGAGCACC
AGCAACAUUUUUACCACCAACUUUGGCUUUAUUAUGAGCCGCAACCGCUUUGAAAUUCUG
AUGCAGAUGCUGCAUUUUAGCGAUAACACCACCGCGGAUAUUACCAACCGCCUGUAUAAA
CUGGGCAGCGUGAUUGAUGAUAUUAUUAUUAACAGCAACCAUUGCAUGCAGCCGCAGGA
A
GAUCUGUGCAUUGAUGAAAGCUUUGUGAAAUUUAUGGGCCGCCAUUGGUAUAGCAGCGU
G
GAACUGGCGGAACAGCUGAAAACCCGCCAGACCCAUCUGGUGGGCACCAUUCGCAGCAAC
CGCAAAAGCAACCCGAAAGAUGUGAUG

>F01_transcript_29213

AUGAAAUACUUCCUAGAAAAUGAAUUUGAAAACAUGUUUUAUAUGCUCAACAACGUUCA
CAAAAAUAAAUUAAAUAUUCUUUAUCUAUUAUUAUUUAAACAAAAUAUUUCAGAAAGCU

UAAUGUUAGGAAGCCAAACAACAACUCCUAUGAGGAAAAGUGAACGUCCAUGCAAUACA
CCAUCAGCAUCACCAGUAGAAAUUAUUUUACUAAAUAAAAAUUGUAGAGUUGAUCCACC
UAACUCUCUAAAUAUUAUUACUAAAGAUAAAAUUGAUCACUUCCCUGUAAUAGAUGAUC
UUAAACAUGGCACUCUAUGUAAAAAUAAAACUUAUAAAAAGAGAACUUGCAUUCAAUAU
AUAAUGUAA

>F01_transcript_31652

AUGAAUAUUAAUUAUGUGAAUUUAGCUGAAUUUAAUAUAGUAUGUUAUGAAGCAAUAUA
UACAUGUAUAGAUAAACCCAAAUUACGUCAAAUUAUUUCUAACCCAGGUUUGAUAAUAG
CGAAAUGUUCACUGAAACAAGUUGAUAUCACAUCCAUGAGUGUAAUGCUAUACCAUCUA
AAUAACUAA

>F01_transcript_32020

AUGGAAUAUUGUCACGGUAUUGAAUCACCUCCAAAAUAUCUCAUUUCAAUUAAUGAAAA
CAGAAUGUGGAAGAUAAAUAAAAACCCAAAUUACAUUAAACCUUCAUUUAGUAUUCAUC
AAGUUCCUAAUGACCAAUUUGCAUCUUCAUCUGGAACAGGUAAACAAUUACGAGACUUC
GACUUGAAUCAAAUACCUUCAGACGAAGAUGAAUAA

>F01_transcript_34671

AUGAAAUACUUCCUAGAAAAUGAAUUUGAAAACAUGUUUUAUAUGCUCAACAACGUUCA
CAAAAAUAAAUUAAAUAUUCUUUAUCUAUUAUUUAAACAAAAUAUUUCAGAAAGCUUAA
UGUUAGGAAGCCAAACAACAACUCCUAUGAGGAAAAGUGAACGUCCAUGCAAUACACCA
UCAGCAUCACCAGUAGAAAUUAUUUUACUAAAUAAAAAUUGUAGAGUUGAUCCACCUAA
CUCUCUAAAUAUUAUUACUAAAGAUAAAAUUGAUCACUUCCCUGUAAUAGAUGAUCUUA
AACAUGGCACUCUAUGUAAAAAUAAAACUUAUAAAAAGAGAACUUGCAUUCAAUAUAUA
AUGUAA

>F01_transcript_35342

AUGUACAAAUUAUUGUUAUUGACAGUCGCGGCAUUGGCUUUGGUAGCCGUCGCUUCCGC
UCAAGUCUUCCUCACUUACCCUCAAACGUACGCGCCGUACUAUACGUACCCGUCGUAUCC
AUACUCGGCUUACCCGUUGACUUACAACCCGUACAUCAGUCUCCUUCGUCGAUAG

>F01_transcript_38899

AUGGGAAAUGAAAAAAUUAAACCGGAACCUAUUGUACAAUACAAUAGAUUUAUGAAUAA
UAUAGAUCGUCAGGGUCAGAUGCAUAAUUAUUACCCUUUCACUAGGAAAACUGUAAGGU
GGUACAAACAAAUCGGGGUGCAUGUCAUACAAAUGUUGCUUAUGAAUUCGUUCUAUUUA
UAUAAUAAACAUUGCAAUGGAUCAAAAAUGUCAUUAUAUGAUUUUCGCAUAUCUGUACU
AAGUGAACUUUUACCUAAACAACCAAAACCAAGAUCGUUGCAAUUAAAAGAGAGCCAUU
UUCCUACAAAAUAUGUAAGCGAAAAUAAUGGAAAAACUCCGAGAAAAAGAUGUCGGUUG
UGCUAUUCGAAUAACAAAACACGGAAGGAUACUUCAUACUAUUGUAUAAGUUGUCCAGG
ACAACCCAGUCUUUGCAUUGAACCAUGUUUUAAAAAUUUUCAUACCAAGCUCCAAUGA

>F01_transcript_51244

AUGAACCUGCCGCCGUGCCCGAGCCGCAACGUGCGCAACGUGAACAAAGUGCUGACCGGC
GAAGAACUGAUUAACAUUCUGGAAAACAGCGAUGAAGAUUUUGGCGUGAGCAGCGAAAG
CGAUCUGGGCGAAGGCGAUAGCGCGGAUGAAGAUAGCAACAGCGUGUUUCAUGAAAACC
CGAUUGUGCAGAACGAUAGCAACACCGUGAACAUUAACCGCCCGGUGGAACGCCAGAGCA
GCGAUUAUACCUGGGUGGAUGAACCGCUGCAGCAUGUGAACAAAGUGCUGUUUAGCAAA
ACCGCGGGCCUGAAAACCCGCCCGAUUGGCAACAAACCGAUUGAUUAUUUUAGCAUGCUG
UUUACCAACGAAUUUAUUGAAUUUAUUGUGCAUGAAACCAACCUGUAUGCGGUGGAAAU
UUUUCUGAGCAUUAGCGGCAGCCUGAGCAGCCGCAUUAGCCAGUGGAAAGAUACCACCGU
GGAAGAAAUGAAAAUUUUUCUGGCGCUGCUGUUUCAUACCGGCACCAUUCGCACCAACCG
CCUGGAAGAUUAUUGGAAAAAAAGCGAACUGUUUAACCUGAACUUUUUUCGCAGCCAUA
UGAGCCGCAACCGCUUUAUGCUGAUUCUGCGCGCGCUGCAUUUUAGCCAUAGCGAAAGCA
GCAACCGCCUGAACAAAAUUGAAAACAUUGUGAACUAUUUUAACAAAAAAAUGGAAGAA
GUGUAUGAACCGAGCAAAAACCUGGCGCUGGAUGAAAGCAUGGUGCUGUUUCGCGGCCG
CCUGGUGUUUCGCCAGUAUAUUAAAAACAAACGCCAUAAAUAUGGCGUGAAACUGUAUA
UGCUGACCGAACCGAACGGCUUUGUGCAUCGCGUGAUGAUUUAUAGCGGCCAGGCGCAUG
AUAUUAGCCUGGAUUGCUGCCAUACCGAAUAUGUGGUGGAAAAACUGAUGGAAGGCCUG
UUUUAUAGCGGCCGCAGCCUGUAUAUGGAUAACUAUUAUAACAGCGUGAAACUGGCGCA
UAACCUGCUGAGCAAAGAUACCUAUUGCACCGGCACCCUGCGCCAGAACCGCAAAUAUAA
CCCGAAAAACGUGAUUAGCAUGAAACUGAAAGUGGGCGAAAACGUGGGCAAAUAUACCG
AAGAAGGCGUGUGCGUGGUGAAAUGGCGCGAUCGCCGCGAAGUGCUGAUGAUUAGCAGC
GAAUUUGAAAACGAAAUGAUUGAAGUGACCAACCGCCGCCGCCAGACCAAACUGAAACCG
AUUGCGAUUAGCAAAUAUAACCAGUAUAUGAGCGGCGUGGAUAAACAGGAUCAGAUGAU
GAGCUAUUAUCCGUGCGAACGCAAAACCCUGCGCUGGUAUAAAAAAAUUGGCAUUCAUU
UUUUUCAGCUGUUUCUGCUGAACAGCUAUUAUCUGUUUAUUAAAUAUGAAAAAAAAAUU
ACCUUUUAUGAUUAUCGCCUGAGCGUGAUUAAAGCCUGUGCGAAAGCAACGUGAUUAG
CAGCCUGCCGCUGCCGACCCAGCGCAAAAGCAUUCAUGUG
>F01_transcript_52179

AUGUUGACUUUUAUAGCGUUAGUCAUUCAUACUGGUACCAUAAAAGUGAACCGGUUGAA
UGAUUAUUGGAAAAAGCACCACCUAUUUGACAUGUCUUGCUUUUCAAAUUACAUGAGCC
GUGAUAGAUUUUUGAUUUUAUUGCGAUGUUUUCAUUUUUCUCCAAAUAUGUUAAAUUGU
GACGGAGAACAGUCUAUAGUUCAUUUAACUAAAAUUAGACCAUUAAUUAAUUAUUUUAA
CAGUAAAAUGAAUUCUAUAUAUUAUCCAAGAAAAGAGUUAUCACUCGAUGAAUCUAUGG
UAUUAUUGAGAGGGCGUUUAAUAUUUCGACGAUAUAUUCAAAAUAAGCGGCAUAAGUAU
GGGAUUAAACUUUAUAUGCUAACCGAGCCAAAUGGAAUAGUUAUAAAAUUUGCAGUUUA
CACAGGAGCUCUUGACGAUAUGGGAGGAAAAGAUCACGCAACUAACGUAGUAAUGCAUU
UAAUGUCAGAAAAAUUAAAUAAUGGUCAUGCUUUAUACAUGGAUAAUUUUUAUAAUACU
UUUGAUUUGGCUACUAAAUUAAUUGAAAAAAAUACGUAUUGUACUGGUACUCUGCGAGU
UAACAUGAAAAAUACACCAAAGGAUGUAGCACAGGCGAAGUUGAAAAAAGAUGAAAAAA
UUGCAAGAUAUUCACAGGGGGUUGUAAUUGGAAAGUGA

>F01_transcript_53440

AUGAAUAGCCAAAAAACUGGAAUGAAGUGCUCAGUUAGGUUUUGUCAAAGUAAAGGUAG
UGAUAGUAAUAAAAGUUUUUUCAAAUACCCCAAUGACGAGCGUCUUGCGACAUGGAUAA
AUAAUUGUAAGACUGGAGACCUAAUCGAAGCACUCAAUAAAAAAAAAAUAG

>F01_transcript_60672

AUGAAAUACUUCCUAGAAAAUGAAUUUGAAAACAUGUUUUAUAUGCUCAACAACGUUCA
CAAAAAUAAAUUAAAUAUUCUUUAUCUAUUAUUAUUUAAACAAAAUAUUUCAGAAAGCU
UAAUGUUAGGAAGCCAAACAACAACUCCUAUGAGGAAAAGUGAACGUCCAUGCAAUACA
CCAUCAGCAUCACCAGUAGAAAUUAUUUUACUAAAUAAAAAUUGUAGAGUUGAUCCACC
UAACUCUCUAAAUAUUAUUACUAAAGAUAAAAUUGAUCACUUCCCUGUAAUAGAUGAUC
UUAAACAUGGCACUCUAUGUAAAAAUAAAACUUAUAAAAAGAGAACUUGCAUUCAAUAU
AUAAUGUAA

>F01_transcript_69130

AUGAAAUACUUCCUAGAAAAUGAAUUUGAAAACAUGUUUUAUAUGCUCAACAACGUUCA
CAAAAAUAAAUUAAAUAUUCUUUAUCUAUUAUUAUUUAAACAAAAUAUUUCAGAAAGCU
UAAUGUUAGGAAGCCAAACAACAACUCCUAUGAGGAAAAGUGAACGUCCAUGCAAUACA
CCAUCAGCAUCACCAGUAGAAAUUAUUUUACUAAAUAAAAAUUGUAGAGUUGAUCCACC
UAACUCUCUAAAUAUUAUUACUAAAGAUAAAAUUGAUCACUUCCCUGUAAUAGAUGAUC
UUAAACAUGGCACUCUAUGUAAAAAUAAAACUUAUAAAAAGAGAACUUGCAUUCAAUAU
AUAAUGUAA

>F01_transcript_70141

AUGCCCGAAAAUGGAACAGAUUUACCUCAUCAAACACUGAUAGAUCAUGGGUUACAUAA
UCCUGCACUCUAUGUAAAUACAAAAAAAAGAAAAAAAGUCUUUAACUUACAAAGUUCUA
AAACUUAUGAGUACAAUGAUUGUUAUUUUAUGUCCAUUUUCAGAAAAAUGAAAAAACUG
UAA

>F01_transcript_73602

AUGGAAAGACUUUUUUAUAGUGGCCGUUCACUUUACAUGAAUAAUUAUUAUAAUAGUGU
UAAAUUAGCACAUAAUCUUCUAAGCAAAGAUAUUUAUUGUACAGGGACUUUAAGACAAA
ACAGGAAAUAUAAUCCUAAGAAUGUAAUUUCGAUGAAAUUAAAAGUAGGAGAGAAUGUU
GGUAAGUACACAGAAGAAGGUGUGUGUGGUAAAAUGGCAAGAUAG

hAT transccript

>F01_transcript_12280

AUGUUUACGGGAACCGGAAGAAAAAAAGAUAUUGUUUGGAAUUAUUUUUCUGAAGUAAA
GACAAAUUCCAAAUCAAAUAGAGCAAAAUGUAACAACUGCGACACUGAAAUGUCAGCUU
UAGUGUCAAGAAUGAAAACGCAUAUAUAUGAAAGAUGUUCAGGAGCUGGAAGUUCAAAU
AAUAUUGAUAAUCCAUCGUCUUCAAAUAUUGACAAUUUACCAGUUAAAAAUGAAAACUC
AGUCCAAGAUAUUUCAGUUAACACGACUUCCACACAUGACAAUCAUGUUUCUUCAGUUA
AUUCAAAUACAACGAUCCAAUUAAAUAAAAAGAAAAAGACUACUCAUCUAUCACCAUCA
UUAAAUACAUUUGUUAUGCGAACAACAGAAGCUGAUAAACAUAAUUUGGAUACCCAAGU
UGCACGAUUUAUUUAUGCAACAAAUACCGCUUUUCGUGCAGUUGAACAUAAAGAGUUUG
UAAAAUUGGUAAAUAUGUUACGUCCUGGUUAUGUUCCACCAAAUAGGCAUUCUAUUUCC
AAUGAAUUAUUAAAUCAGGUUUAUGAUUCUCUCACUAGCUUAAAUAAACAAGAGUUGGA
AGGAAAGACAGUUUGUAUGGCAUUGGACGGUUGGUCUAAUGUUCAUAAUGAGUCCAUUA

UUUGUGUUUGUGUAACCGAUAUUGAUGGGGAUUUGGUUUAUUUAUUAGACACAAUAGAU
ACAAAAGACAAUAGUCAUUCGUGGGAUUAUUUAGUAAAUUUAGUAGAGUCUUCUAUCAA
AUUUUGUGAAACUUUUGGUUGUACUGUUGGCAGUGUUGUUACUGAUAAUGCUGCCAAUA
UGAAUAAAAUGAGAAAGAAUCUAGCCACUUGUGAAGGUUUAAAAAAUAAACAUGUAAUU
ACAUUAGGAUGUUCAGCACACUUACUAAACUUGUUAGCGCAUGACAUAGAAAUCCCUGG
UGUCAAAAGUCAUAUAAAAACAAUAUUUAAAUAUUUUAAAAAUUCUCAUUUUUUUGGAG
CCAGGUAUAAAUUAGAAGGAGGAAAGGCAUUAGUUCUUCCUCAAGAUAUUCGAUGGAAU
ACACUUGCUGAUACUAUACAAUGUUAUUUGAAUAACUGGCAUAUAUUAUAUAAAGUUUG
CAAUGAUAAUCGUGCAGCCAUUGAUGAAACUAUUUUUAUUAAAGUUAAAGAUCUACAUA
UUAAAUCUAUGGCCCAAGAAUAUUUGGUGAUCCUUAAAAAAGUAGCUAUAGCAUUAGAC
UUAAUGCAGGCAGAUAGUUGCACCAUUAGUGAGGCAACUCAUAUAUGGUUAGAUCUAAA
ACGUUUUUUUGAAAUUGAGACACAUAAUUCUUCAAUGAUCGAUUUAUUCAAUUAUAGGU
UUGAUAUGGCAAUAACUGAGUAUCAUUUAUUAGCUUAUAUUUUAGAUCCUAGAUAUUUU
GGUACAAAACUGACCGAUGACCAAUUAGACUCUACUUUAAAUUUUGUAAAUAUAUAUCA
CCAAGAGAUAAUGUCUGAAAUUAUAAAAUUUCAAGCUCGUUCAUUUCCAUUCAAAGAAU
AUCUUUUUUCUGAAAGCACAGUUAAAAAUAUUAAACCAUUAACUUGGUGGUUAGCCUUA
AAAAAUACAAUUUCACACGAAAUGUUAGAAUUGGUUAUAAAACUAUAUACAGCUGUAGC
UUCUUCGGCGGGCAUAGAACGUAUUUUUUCAACGUAUGGUUUAGUUCAUUCCAAAUUAA
GGAAUAGGUUAGGAAUUGAAAAAUCUUCCAAAUUAGUAGCUAUUUUUAAAUCCUUAAAU
AAACAUUGUGAUAAUAAAAUUGAUGAUUAA

>F01_transcript_23556

AUGUUUACGGGAACCGGAAGAAAAAAAGAUAUUGUUUGGAAUUAUUUUUCUGAAGUAAA
GACAAAUUCCAAAUCAAAUAGAGCAAAAUGUAACAACUGCGACACUGAAAUGUCAGCUU
UAGUGUCAAGAAUGAAAACGCAUAUAUAUGAAAGAUGUUCAGGAGCUGGAAGUUCAAAU
AAUAUUGAUAAUCCAUCGUCUUCAAAUAUUGACAAUUUACCAGUUAAAAAUGAAAACUC
AGUCCAAGAUAUUUCAGUUAACACGACUUCCACACAUGACAAUCAUGUUUCUUCAGUUA
AUUCAAAUACAACGAUCCAAUUAAAUAAAAAGAAAAAGACUACUCAUCUAUCACCAUCA
UUAAAUACAUUUGUUAUGCGAACAACAGAAGCUGAUAAACAUAAUUUGGAUACCCAAGU
UGCACGAUUUAUUUAUGCAACAAAUACCGCUUUUCGUGCAGUUGAACAUAAAGAGUUUG
UAAAAUUGGUAAAUAUGUUACGUCCUGGUUAUGUUCCACCAAAUAGGCAUUCUAUUUCC
AAUGAAUUAUUAAAUCAGGUUUAUGAUUCUCUCACUAGCUUAAAUAAACAAGAGUUGGA
AGGAAAGACAGUUUGUAUGGCAUUGGACGGUUGGUCUAAUGUUCAUAAUGAGUCCAUUA
UUUGUGUUUGUGUAACCGAUAUUGAUGGGGAUUUGGUUUAUUUAUUAGACACAAUAGAU
ACAAAAGACAAUAGUCAUUCGUGGGAUUAUUUAGUAAAUUUAGUAGAGUCUUCUAUCAA
AUUUUGUGAAACUUUUGGUUGUACUGUUGGCAGUGUUGUUACUGAUAAUGCUGCCAAUA
UGAAUAAAAUGAGAAAGAAUCUAGCCACUUGUGAAGGUUUAAAAAAUAAACAUGUAAUU
ACAUUAGGAUGUUCAGCACACUUACUAAACUUGUUAGCGCAUGACAUAGAAAUCCCUGG
UGUCAAAAGUCAUAUAAAAACAAUAUUUAAAUAUUUUAAAAAUUCUCAUUUUUUUGGAG
CCAGGUAUAAAUUAGAAGGAGGAAAGGCAUUAGUUCUUCCUCAAGAUAUUCGAUGGAAU
ACACUUGCUGAUACUAUACAAUGUUAUUUGAAUAACUGGCAUAUAUUAUAUAAAGUUUG
CAAUGAUAAUCGUGCAGCCAUUGAUGAAACUAUUUUUAUUAAAGUUAAAGAUCUACAUA
UUAAAUCUAUGGCCCAAGAAUAUUUGGUGAUCCUUAAAAAAGUAGCUAUAGCAUUAGAC
UUAAUGCAGGCAGAUAGUUGCACCAUUAGUGAGGCAACUCAUAUAUGGUUAGAUCUAAA

ACGUUUUUUUGAAAUUGAGACACAUAAUUCUUCAAUGAUCGAUUUAUUCAAUUAUAGGU
UUGAUAUGGCAAUAACUGAGUAUCAUUUAUUAGCUUAUAUUUUAGAUCCUAGAUAUUUU
GGUACAAAACUGACCGAUGACCAAUUAGACUCUACUUUAAAUUUUGUAAAUAUAUAUCA
CCAAGAGAUAAUGUCUGAAAUUAUAAAAUUUCAAGCUCGUUCAUUUCCAUUCAAAGAAU
AUCUUUUUUCUGAAAGCACAGUUAAAAAUAUUAAACCAUUAACUUGGUGGUUAGCCUUA
AAAAAUACAAUUUCACACGAAAUGUUAGAAUUGGUUAUAAAACUAUAUACAGCUGUAGC
UUCUUCGGCGGGCAUAGAACGUAUUUUUUCAACGUAUGGUUUAGUUCAUUCCAAAUUAA
GGAAUAGGUUAGGAAUUGAAAAAUCUUCCAAAUUAGUAGCUAUUUUUAAAUCCUUAAAU
AAACAUUGUGAUAAUAAAAUUGAUGAUUAA

>F01_transcript_40427

AUGUUGUUGCAAUUGCAACUUUUUCCCCAUUGUUUAGAUUUGUUGCUAAGUAUGUUUUU
UAAAUUCGUUUCUCGGGAGUACGAAUUAAAAUCUAAAAGUUUUUGGUGCAAUUUUUUGC
AAUUUGAGGCAUUUUUUCAAUUUAAUUGCAAUUUUGUGAAUUUUUUCAAUUUAAUUGCA
AUUUUGUCGAUUUUUCUCAUUUAUAGUACAAUUUUAUUUACCUAUUUAUGUCAAUUAUU
GGGAGUAAUAUAUAUAUAUUUUUUUAUAGUUAUUUUUAUAUUAGGACAUAUUGUAUGCG
UAUUGAUGUCCAUUUCGAGUCAGCUGUUAAGGUCUGCUAUUAAUAAUAAAAUAUUAAAU
AAUGAUUAUUUUCCCAAAAUAGUAGUAUUUCAGUAUAUUUUUACAGAAUAUUAUAUUAG
UGUCGUUCUAUCUAUCGUCGCGCAUUUAAUAAAAUUGUACGUAUUACGUUUGCUGGCCG
UUCAGAAUAAAUUCUUGUCUAAAAUGCCGAAAAUUAAGCCGUCGAUGACUAGUGUUUUA
UCUAAAUAUGUCAGUGAGUUCGGGGAAAAUGUUUUUUCAAGUAAGGAGUCCAUUCUUUU
UUGUAAAUUGUGUGAAGUUCGAGUGUCUGCAGAUCGAAGAUAUAUAGUAACACAACACU
UAAAAACUGACAAACACAUUCGCGCAAUAAAUCGCAAUAAAAACGAAACAACGUCAAAA
GUACAGCAACAACUUACGCUGUCCUCAAAAAAAACUACGUUUUCCAAAGAUCAUUGUAA
AGCUUUAUUAUCUGCAAACAUUCCUCUAAACAAAGUCAACAAUAAGGAUUUCCGUUUGU
UUAUGGAGAAAUACACGAACAGAGAAAUUCCUGACGAAAGCACGUUGCGUAAAAGUUAC
GUGAACGAUAUUUACGAAGAAGUUAUUAACAAAAUCAGAUUUAAUAUUGCUGGACACAA
AAUAUGGGUUUCGGUGGAUGAAACCACCGAUGUUCAAGGCAGAUAUAUUGCUAAUAUUA
UUAUUGGAACAUUAGAAGUAGACAAUCCUGGUCAAGUUUACCUUUUGAACUCAGAGGUG
CUCGAGAAGACAAAUUAUUCGACAAACACAAAAGUAUUUGACCGAUCGAUGUUUCUACU
UUGGCCUGGUGGUAUACGCUAUGAUGACGUAUUACUAUUUGUCAAUGAUGCGGCGCCAU
ACAUGAUCAAAGCCGGAAAAUCAAUUGGAGUACUCUAUUCUAAGAUGGUGCAUAUUACG
UGCCUAGCACAUGGUGUACAUCGAGUUGCUGAAGAAAUAAGAGGAAGAUUUGCUAAUGA
UGAUAAAUUGAUUUCCAAGAUAAAACAAAUAUUUUUAAAAUGUCCAGCCCGUGUAUUAU
UUUUUAAAAAUAAAGCUCCAAAUAUACCAUUACCUCCCCAAUCCAUGGUCGCCCCGAGGG
GUGGUGGCAAGGCGGGGCAUCUGCCCCCCCUGGACUUCAAAACUUCAAAUAUAUACUCUG
AAAUUUUUUAA

>F01_transcript_45682

AUGAGAGUAUAUGAGUUGUCUACUGUAGUAUUUUCUGUGCCUGGAACACAAUUCAGUGU
AGAGAGACUUUUUUCUGGUCUUAAAUUCAUUUUAUCACCUUACAGAAGUAUUACUAAUG
CACAUAAUUUGGAGGAUCAAUUAAUUGUGCAGAUAAAUAGGAAGUUUUGA

>F01_transcript_58521

AUGAUUUUUAAAAUCUUAUAUUUGAAAACACAAAUUUACUAUCGUAUUCUGGACAUAAU
UAUUGUACAACUUAAACACCGUUUUAAUGGUAUGAACCAAGUAAUAAAACUUUUUACUU
CAAUAAUACCAAUUAAUAUAUGUUCAAUGAAUGAAAAUGUACUUAAACAUUCAACUACC
UUUUUAGUAAAUAAUUAUGAAAAUGAUUUAACAAUAUAUUUGGUCAAUCGAAUUAUUCA
ACUUAAACGUUCGUUUGAAAAUCAAAUUUCAAAAUUAAAUUCUAUAGAUGACUUGGCAA
CAUUUUUAAUUGUAGACAAUUAUUUAAUAGCGGCAAAUUUUCCAGAUUUAUGUACAACU
UGUUUUCUUUUUUUAACAAUACCAGUGACGGUGGCUAGUGCAGAAAGACGUUUUUCUAA
ACUUAAAAUAAUUAAAAACUGUCUUCGAAGUACGAUGUCCCAAAUCCGUUUGUCAAGUU
UAGCUAUAAUAUCAAUAGAAAAGAAAAUUGCCAAAGAAAUUAAUGCAUCAGAUAUUAUU
UCUACUCUAGCAAAUAAGAAGUCCAGAAAAAUGUUUUAA

>F01_transcript_71708

AUGGUUAAAAUUAUUGAACAAGAUGCAUUGAUAGACAUUUUAUCUGAAGUUUAUAAAUU
AUUUAUUCUUAUUUUAACCAUACCAUCAAUUAAUGUAUCAAAUGAAAGAAGUUUCUCUU
GCCUCAAAAGAACCCUGAAAUAUUUAUCAAAAAUCAAUAUGUAA

>F01_transcript_79136

AUGCGAACAACAGAAGCUGAUAAACAUAAUUUGGAUACCCAAGUUGCACGAUUUAUUUA
UGCAACAAAUACCGCUUUUCGUGCAGUUGAACAUAAAGAGUUUGUAAAAUUGGUAAAUA
UGUUACGUCCUGGUUAUGUUCCACCAAAUAGGCAUUCUAUUUCCAAUGAAUUAUUAAAU
CAGGUUUAUGAUUCUCUCACUAGCUUAAAUAAACAAGAGUUGGAAGGAAAGACAGUUUG
UAUGGCAUUGGACGGUUGGUCUAAUGUUCAUAAUGAGUCCAUUAUUUGUGUUUGUGUAA
CCGAUAUUGAUGGGGAUUUGGUUUAUUUAUUAGACACAAUAGAUACAAAAGACAAUAGU
CAUUCGUGGGAUUAUUUAGUAAAUUUAGUAGAGUCUUCUAUCAAAUUUUGUGAAACUUU
UGGUUGUACUGUUGGCAGUGUUGUUACUGAUAAUGCUGCCAAUAUGAAUAAAAUGAGAA
AGAAUCUAGCCACUUGUGAAGGUUUAAAAAAUAAACAUGUAAUUACAUUAGGAUGUUCA
GCACACUUACUAAACUUGUUAGCGCAUGACAUAGAAAUCCCUGGUGUCAAAAGUCAUAU
AAAAACAAUAUUUAAAUAUUUAAAAAUUCUCAUUUUUUUGGAGCCAGGUAUAAAUUAG
AAGGAGGAAAGGCAUUAGUUCUUCCUCAAGAUAUUCGAUGGAAUACACUUGCUGAUACU
AUACAAUGUUAUUUGAAUAACUGGCAUAUAUUAUAUAAAGUUUGCAAUGAUAAUCGUGC
AGCCAUUGAUGAAACUAUUUUUAUUAAAGUUAAAGAUCUACAUAUUAAAUCUAUGGCCC
AAGAAUAUUUGGUGAUCCUUAAAAAAGUAGCUAUAGCAUUAGACUUAAUGCAGGCAGAU
AGUUGCACCAUUAGUGAGGCAACUCAUAUAUGGUUAGAUCUAAAACGUUUUUUUGAAAU
UGAGACACAUAAUUCUUCAAUGAUCGAUUUAUUCAAUUAUAGGUUUGAUAUGGCAAUAA
CUGAGUAUCAUUUAUUAGCUUAUAUUUUAGAUCCUAGAUAUUUUGGUACAAAACUGACC
GAUGACCAAUUAGACUCUACUUUAAAUUUUGUAAAUAUAUAUCACCAAGAGAUAAUGUC
UGAAAUUAUAAAAUUUCAAGCUCGUUCAUUUCCAUUCAAAGAAUAUCUUUUUUCUGAAA
GCACAGUUAAAAAUAUUAAACCAUUAACUUGGUGGUUAGCCUUAAAAAAUACAAUUUCA
CACGAAAUGUUAGAAUUGGUUAUAAAACUAUAUACAGCUGUAGCUUCUUCGGCGGGCAU
AGAACGUAUUUUUUCAACGUAUGGUUUAGUUCAUUCCAAAUUAAGGAAUAGGUUAGGAA
UUGAAAAAUCUUCCAAAUUAGUAGCUAUUUUUAAAUCCUUAAAUAAACAUUGUGAUAAU
AAAAUUGAUGAUUAA

Copia transcripts

>F01_transcript_60360

AUGGCAGUUGGCAAAGGUAACAUAAAUGUUCAUGCUUAUGUUAAUAAUAAGUGGGUAGA
CCGUUAUUUAGCAAAUGUUUUACAUGUGCCGGAUCUUAAAGUAAAUUUAUUUUCGUGUG
GGGCAUGUCUCGAUAAGGGAAUUGAAAUGAUGACAAACAGAAACGGGUGUACGUUUAAA
AGAAAUAAUCGUGUAAUUGCAGUCGGUGUGCGUGAGAAAAAAUUAUUUUCAAUGUUAAU
CAAAGUCGAUAGUUCACAGGAAAUCGAACAUGCAAACAUUGCAGUUAAGAAUUACACAU
UACAACACUGGCACAACGUGCUCUGUCAUCAGAAUGUCAAACAUGUUCGUGAGUAUUUG
AAACAUAAUAAAAUACAAUUUACAGAGACACAAGAACAGUUUUUUUGUGAACCGUGCAU
UUAUGGUAAACAGCAUAAAGAGCCAUUUACACAAAGUGUAACAAUAACAACUGAACCUG
GUCAAAUAAUUCAUACUGACGUAUGUGGGCCAAUGGAAGAAAACUCGUUAGGAGGUAAA
AGAUAUUUUGUUAUUUUUAAAGAUGAUUAUUCCAAAUAUACAUAUGUAUAUUUUAUAAA
ACACAAAUCUGAGGUAAAAGAAAAACUUAAACUUUUCCUAAAUACAGUUAAUACUCAAA
CAAAUAUUGUUGUUUAA

>F01_transcript_67873

AUGGACUUAUCUAGUUUACUAUCGAUCAACAUGGAUCUUGCACGUUCAACAAUUGUUCU
AUUUAAACGUUCCGUAUGUCCAUUCAUUUCAGCAGUAUAUGGAGCAGUGUAUUGUAAUU
UUAUACCUUUUUGUUUACAAAAAUUAGUAAGUACUUUAGAUGUGUAUUCACCACCAUUA
UCACAUCUUAAAGUUGAAAUACAAAUAUUGAAUUUCGCACUUACUUAA

Helitron transcripts

>F01_transcript_21216

AUGGAAAUUCUAACGGUCAAAUUUACAGCGAGUUCACGGUACAUGAAUGAAUACGUACA
AGAUACAAUGACAUACAUUCGAGCAUAUGGCCGCCCAGAUUUGUUCAACACUUUCACAUA
CAAUUCUAUGUGUGAUGAAAUGAAAGAACUAGUGUUUCUAGCAAACAAAAGUUCUUUAA
UUCAAUCAUCAUCAGAUCGUCAUGAUAUUACCACGCGCGUAUGCAAACAAAAAUUAAAA
UCUUUUAUAGAUUUUAUUGCAAACCAACGUGUUUUUGACGAUACACGUUAUUAG

>F01_transcript_32632

AUGUCGACAAUUAUUUCAUUAAUAUAUUGUCAUAUGCUUGCACAAAUCGAAACGGAACA
AUUUCUUUUCAUUCGGUUGAAUCAAACCAAGUUGUGUUCAGAAGAAUAUAUACAUUUAC
GUGAUACAGUAGCCAACGAUGACAAUAUGAAUCCCAAUGCAUUGGAAAAAAUGGUAAUU
UUACCAGCUAUAUUUACAGGGAGUCCACGGUACAUGCAUGAAUAUGCACAAGAUGCAAU
GACAUACGUCCGAGCAUAUGGUUUCCUAGAUUUGUUUGUCUACGCACUAGAAGGAAAAA
CAAAAAAUAUUAUGUAUCCUAAAGCACUUCAAUAA

>F01_transcript_37515

AUGCACGAACGAGCCCAAGAUGCACUGACGUACGUUCGACAUUAUGGCAGUUCAGAUUU
AUUCGUAACUUUUACGUGUAAUCCAAAAUGGCAAGAAAAUCCAAAAAGCACUUUUACCU
GGACAAAAACAUUACCAUCGCCCGGACAUCAUUGCAAGAGUUUUUAA

>F01_transcript_47477

AUGUUUUGUUAUGGAAAAGAUGGAUACUCAAUAGAUAAUUCUCAGACGGAUGCAACUAC
AAAAUUUCCUCUUAAAGAUAAAACUGUAUUUGCUGUAAAUUUUUAUUCAUACAGAAUUA
UGUUAAGGAAAGAACUAAAUAAUCAUUUGCUUCGUUAUGGUCCAUUGUUCAACCAAUAU
UUAGUAGAUAUGUACGCAAAAAUAGAGACUGAAAGAUUAAAUUUCAUCAGAAAUCAUCA
AAAUAAGCUUAGAGCAGAUAAUUAUAUUAAUUUAAAAGAUGCCGUUGGAAGACAAGAUG
UUGAAGCAGAUCAACUUGGAAAAUUAGUUGUACUGCCAUCAUCUUUUAUAGGGGGUCCA
UGA

>F01_transcript_48429

AUGGUCAUACUUCCGGCUACAUUUAGAGGAAGCCCACGACAUAUGCAUGAAUACGCACAA
GAUGCAAUGACUUACGUUCGCGCAUACAGUUAUCCAGAAUUUUUCAUAACAUUCACGUG
CAAUCCUACGUGGCAUGUAACUAUAGAACUUCAGUUGCCUAGACAAUUAUUUUCGGAUC
ACCAUGACAUAAUUGCACGUGUGUUCAAACAAAAAUUGAAAUCUUUUAUAAAUUUCAUU
UACGAGUAUCGUGUUUUUGGUGAGACACGUUGUUGGAUGUAUUCCAUCGAACGGCAGAA
AAAAGGAUUGCUGCAUGCACACGUUUUGAUUUGGUUAAUUCAUAGAAUUACACCAGACA
AAAUUAAUGCAGUCAUCUCAGCAGAAAUACCAGACCGUCGACAUUAA

>F01_transcript_51021

AUGUCGACAAUUAUUUCAUUAAUAUAUUGUCAUAUGCUUGCAAAAAUCGAAACGGAACA
AUUUCUUUUCAUUCGGUUGAAUCAAACCAAGUUGUGUUCAGAAGAAUAUAUAUAUUUAC
GUGAUACAGUAGCCAACGAUGACAAUAUGAAUCCCAAUGCAUUGGAAAAAAUGGUAAUU
UUACCAGCUAUAUUUACAGGGAGUCCACGGUACAUGCAUGAAUAUGCACAAGAUGCAAU
GACAUACGUCCGAGCAUAUGAUUUCCUAGAUUUGUUUGUCUACGCACUAGAAGGAAAAA
CAAAAAAUAUUAUGUAUCCUAAAGCACUUCAAUAA

>F01_transcript_56571

AUGUUUUGUUAUGGAAAAGAUGGAUACUCAAUAGAUAAUUCUCAGACGGAUGCAACUAC
AAAAUUUCCUCUUAAAGAUAAAACUGUAUUUGCUGUAAAUUUUUAUUCAUACAGAAUUA
UGUUAAGGAAAGAACUAAAUAAUCAUUUGCUUCGUUAUGGUCCAUUGUUCAACCAAUAU
UUAGUAGAUAUGUACGCAAAAAUAGAGACUGAAAGAUUAAAUUUCAUCAGAAAUCAUCA
AAAUAAGCUUAGAGCAGAUAAUUAUAUUAAUUUAAAAGAUGCCGUUGGAAGACAAGAUG
UUGAAGCAGAUCAACUUGGAAAAUUAGUUGUACUGCCAUCAUCUUUUAUAGGGGGUCCA
UGA

>F01_transcript_63324

AUGUAUGCAAAAAUCGAAACCGAACGACUUCUCUUCAUUCGGUUGAAUCAAACCAAACU
GUGCUCGGAAGAAUAUAUUCAUUUCCGCGAUGCGAUUGCUACUGAUGGCAAUCCUAAUG

AACUGGGAAAAAUGGUCAUACUUCCGGCUACAUUUGGAGGAAGCCCACGACAUAUGCAU
GAAUACGCACAAGAUGCAAUGACUUACGUUUACGCAUACGGCCGUCCGGAUAUUUUCAU
AACAUUCACGUGCAAUCCUACGUAG

>F01_transcript_75309

AUGCGAAUCUUGCCGUGCAUUGAAAUUCAAAAACGAAACUCCAGGAAUGUGCUGCGCAA
GUGGAAAUGUGAAAUUGCCAGAAUUGUCAUAAUUCCGGCUAUAUUUGGAGGAAGCCCAC
GACAUAUGUAUGAAUACGCACAAAAUGCAAUGACAUACGUUCCCGCAUACGGCUGUCCGG
AUUUUUUCAUAACAUUAACGGUCAAUCCUACGUGGGAUGAAACUAGAGAACUUCUGUUG
CCUGGACAAUCAUCUUCGGAUCGUCAUGACAUCAUUACACGUGUGUUCAAACAAAAAUU
GAAAUCUUUUAUGGGUUUCAUUUACAAGUAUUGUGUUUUUGGGGAGACACGUUGUUGUU
AG
