# Supplementary material for: Mobilome of the Rhus Gall Aphid Schlechtendalia chinensis Provides Insight into TE Insertion-Related Inactivation of Functional Genes
Source: Int J Mol Sci. 2022 Dec 15;23(24):15967. doi: 10.3390/ijms232415967 (PMC9783078; doi:10.3390/ijms232415967)
Supplement: Supplementary file 1 [file ijms-23-15967-s001.zip › Supplementary Figures.pdf]

Supplementary Information

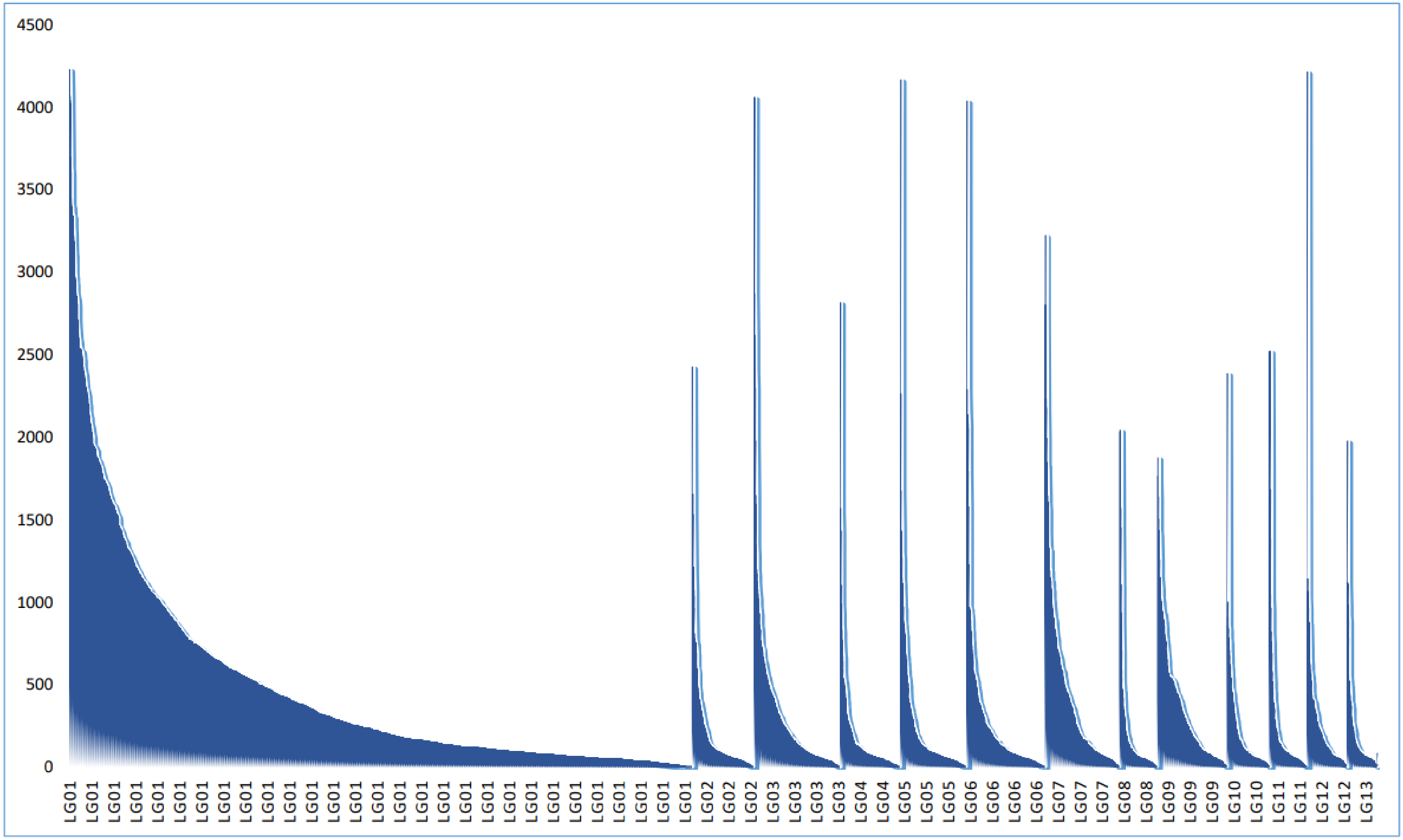

Figure S1. Graph showing length wise distribution of Helitron on the chromosomes of S. chinensis. X-axis represent the number of chromomsomes while the Y-axis reprsent of the Helitron elements.

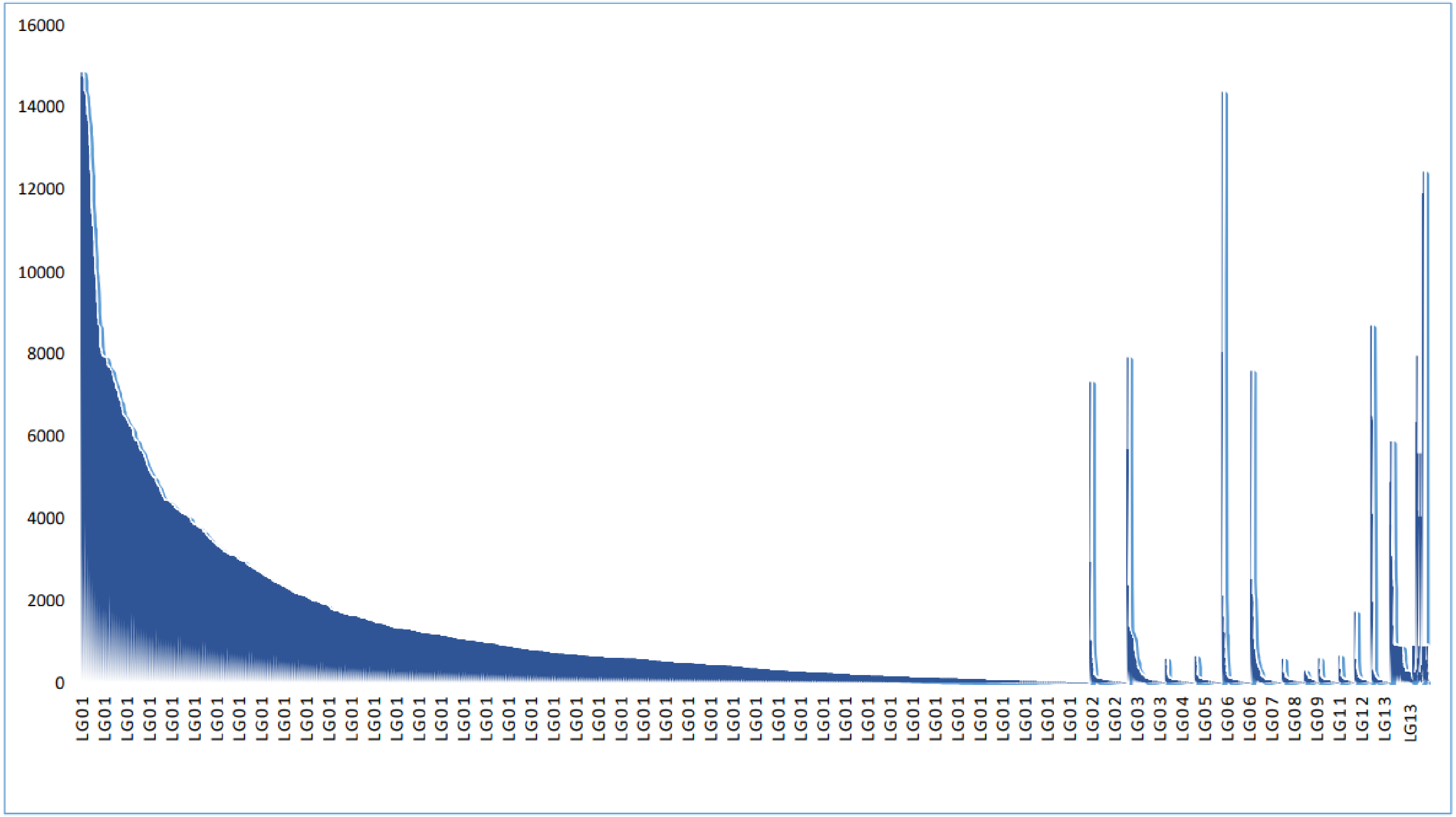

Figure S2. Graph showing length wise distribution of Mavericks on the chromosomes of S. chinensis. X-axis represent the number of chromomsomes while the Y-axis reprsent of the Maverick elements.
